# A Dynamic Time Warping Extension to Consensus Weight‐Based Cachexia Criteria Improves Prediction of Cancer Patient Outcomes

**DOI:** 10.1002/rco2.107

**Published:** 2025-01-29

**Authors:** Noah Forrest, Steven Tran, Khizar R. Nandoliya, Ethan J. Houskamp, Tomasz Gruchala, Vijeeth Guggilla, Zequn Sun, Rimas Lukas, Derek Wainwright, Al'ona Furmanchuk, Jodi L. Johnson, Ishan Roy, Theresa L. Walunas

**Affiliations:** ^1^ Center for Health Information Partnerships Northwestern University Feinberg School of Medicine Chicago Illinois USA; ^2^ Department of Neurological Surgery Northwestern University Feinberg School of Medicine Chicago Illinois USA; ^3^ Department of Physical Medicine and Rehabilitation Northwestern University Feinberg School of Medicine Chicago Illinois USA; ^4^ Department of Preventive Medicine Feinberg School of Medicine, Northwestern University Chicago Illinois USA; ^5^ Department of Neurology Northwestern University Feinberg School of Medicine Chicago Illinois USA; ^6^ Department of Cancer Biology, Stritch School of Medicine Loyola University Chicago Maywood Illinois USA; ^7^ Department of Neurological Surgery Loyola University Medical Center Maywood Illinois USA; ^8^ Department of Medicine Northwestern University Feinberg School of Medicine Chicago Illinois USA; ^9^ Department of Medical Social Sciences Northwestern University Feinberg School of Medicine Chicago Illinois USA; ^10^ Department of Pathology Northwestern University Feinberg School of Medicine Chicago Illinois USA; ^11^ Department of Dermatology Northwestern University Feinberg School of Medicine Chicago Illinois USA; ^12^ Robert H. Lurie Comprehensive Cancer Center Northwestern University Feinberg School of Medicine Chicago Illinois USA; ^13^ Department of Physical Medicine and Rehabilitation Shirley Ryan AbilityLab Chicago Illinois USA

**Keywords:** cancer cachexia, dynamic time warping, electronic health records

## Abstract

**Background:**

Cachexia is a complex syndrome that impacts up to half of patients with cancer. Criteria systems have been developed for the purpose of diagnosing and grading cachexia severity in clinical settings. One of the most widely known is those developed by Fearon et al. in 2011, which utilizes body mass loss and body mass index (BMI) to determine the presence and extent of cachexia. One limitation of this system and other clinical cachexia scales is the lack of systematic methods for assessing cachexia severity longitudinally. We sought to develop an extension to the 2011 consensus criteria that categorizes cancer patients with respect to their temporal cachexia progression and assess its predictive capacity relative to the current time‐agnostic system.

**Methods:**

Two cancer cohorts were identified in electronic health record data: lung cancer and glioblastoma. We extracted weight and BMI measures from the time of cancer diagnosis until death or loss to follow‐up and computed cachexia severity according to the consensus criteria. Subgroups of cachexia progression were uncovered using dynamic time warping (DTW) followed by unsupervised clustering. This system and baseline consensus criteria measurements were each assessed for their ability to stratify patient outcomes utilizing Kaplan–Meier curves and Cox proportional hazards and subsequently compared with model concordance and inverse probability of censoring weighting (IPCW).

**Results:**

Significant differences were observed in overall survival Kaplan–Meier curves of 1023 patients with lung cancer when stratified by baseline cachexia classification (*p* = 0.0002, N events = 592) but not in a cohort of 545 patients with glioblastoma (*p* = 0.16, N events = 353). DTW uncovered three patterns of cachexia progression in each subgroup with features described as ‘smouldering’, ‘rapid with recovery’ or ‘persistent/recurrent’. Significant differences were observed in Kaplan–Meier curves when stratified by cachexia longitudinal patterns in lung cancer (*p* < 0.0001) and glioblastoma (*p* < 0.0001). Adjusted hazards ratios comparing the ‘persistent/recurrent’ cluster to referent subgroups in Cox models were 4.8 (4.1–5.8, *p* < 0.05) and 1.9 (1.4–2.4, *p* < 0.05) among patients with lung cancer and glioblastoma, respectively. Areas under the curve at multiple time points and Cox model concordances were greater when patients were stratified by progression pattern compared with baseline consensus criteria.

**Conclusions:**

Our results suggest that accounting for cachexia's longitudinal progression in a systematic way can improve upon the prognostic capacity of a widely used consensus criteria set. These findings are important for the future development of systems that recognize concerning patterns of cachexia progression in clinical settings and aid clinicians in cachexia‐related decision making.

## 1 Introduction

Cachexia is one of the most common and serious cancer syndromes, with up to 50% of patients affected and approximately 20% of cancer‐related deaths attributed to its sequelae [1]. Cachexia can be diagnosed and graded clinically using one of many criteria sets or clinical and laboratory markers that serve as heuristic guides for clinical decision making for cachexia interventions [2, 3, 4, 5, 6]. The Fearon consensus criteria, established in 2011 [2] is the most widely adopted for cachexia diagnosis and relies on the percent of body weight lost as well as body mass index (BMI) [2]. A strength of this system is that body weight and BMI measurements are reliably documented and easily obtained with inexpensive equipment universally present in clinical settings. However, consensus criteria and other emerging metrics are limited in that they consist of measurements taken at discrete time points during a dynamic disease. Although clinicians may monitor a patient's cachexia severity through longitudinal observation, prognostic information could go unnoticed by clinical intuition alone, highlighting the importance of developing systematic methods for monitoring cachexia during cancer care.

Evidence from both animal models and human studies suggest cachexia's temporality may serve as a valuable prognostic indicator. For example, significant differences in longitudinal metabolite trajectories were detected between animals in a mouse model of cachexia several days before differences in body weight or muscle mass were observed [7]. Repeat abdominal computed tomography images revealed that patients with the highest rate of muscle mass loss exhibited significantly worsened survival compared with individuals with slower loss of muscle mass in metastatic colorectal cancer [8]. We previously reported that single measurements of body composition and blood‐based cachexia markers collected at the time of treatment initiation were not congruent in their ability to predict clinical and functional outcomes in a cohort of patients with B‐cell non‐Hodgkin lymphoma [9]. These observations suggest that measurements of cachexia markers at single time points may provide only limited information and accounting for their progression over time may result in better prediction of patient outcomes.

Characterizing the longitudinal trajectory of cachexia in real‐world patient populations requires measurement of cachexia markers over the disease course [10], and few resources have the required degree of longitudinal depth as is found in electronic health records (EHR). EHR data have been leveraged to characterize many disease processes [11], including an EHR‐based cachexia detection algorithm [12]. In addition, researchers have successfully leveraged this data to identify longitudinal disease phenotypes with the aim of predicting patient outcomes [13]. Because clinical measurements are not uniformly made at the same time points for each patient, one strategy has been to align analogous clinical measurements temporally to compare data sequences between patients [14]. One algorithm developed to address the issue of comparing temporal sequences of events across differing time periods and measurement frequencies is called dynamic time warping (DTW) [14]. DTW has been used in combination with unsupervised machine learning to identify trajectories of comorbidity burden accrual [15, 16] and sepsis severity [17] using data derived from EHRs or insurance claims. An advantage of this approach is that DTW does not make any assumptions about the underlying data, a limitation of other approaches for trajectory identification such as group‐based trajectory modelling [18].

In this study, we test the hypothesis that improved cancer patient prognostic information can be gained by identifying consensus criteria temporal patterns via DTW and unsupervised clustering. We first tested this methodology in a lung cancer cohort because cachexia is highly prevalent in lung cancer and its effect on outcomes is well understood [19, 20]. We then assessed the applicability of our approach to patients with glioblastoma (GBM), a cancer in which limited evidence exists on the prognostic value of cachexia measures [21, 22, 23]. In this work, we show that outcomes reflecting mortality, morbidity and function in both lung cancer and GBM are more accurately predicted when accounting for longitudinal patterns in cachexia criteria measures when compared with a single measure.

## 2 Methods

### 2.1 Data Source and Study Subjects

Patient weight data were extracted for all individuals matching our inclusion criteria from the Northwestern Medicine Enterprise Data Warehouse (NMEDW) between 1 January 2012 and 22 November 2022. The NMEDW is a diverse repository of data elements representing clinical care that patients receive at Northwestern Medicine clinics and hospitals. All patients included in this study were 18 years or older at the time of cancer diagnosis. This study was approved by the Northwestern University IRB protocols STU00217041 and STU00210502. Inclusion in this study required that patients had previously signed a consent form for data use at Northwestern Medicine.

### 2.2 Lung Cancer Cohort

Patients with lung cancer were identified using International Classification of Disease Codes (ICD) Versions 9 and 10 [24, 25] that specified a lung malignancy followed by treatment with immune checkpoint inhibitor (ICI) therapy to identify a cohort under similar therapeutic conditions. The presence of cachexia was previously found to be associated with decreased overall survival in lung cancer patients receiving ICI therapy [19], which may be due to increased catabolic clearance of monoclonal antibodies [20]. This suggests that an interplay between ICI therapy and cachexia may exist. Patients were retained in the analytic cohort based on two data availability criteria. The first required that patients have a baseline weight and BMI measurement up to 6 months prior to cancer diagnosis. The second required that patients had two or more weight measures on or following cancer diagnosis.

### 2.3 GBM Cohort

GBM patients were identified using the most recent (2021) World Health Organization (WHO) definition of GBM including the absence of isocitrate dehydrogenase mutations (IDHwt) [26]. Narrative pathology documents containing the case‐insensitive text string ‘glioblastoma’ were identified from a cohort of patients with a diagnosis code indicative of a brain malignancy. From this set of documents, 1500 (80%) were randomly selected to attain a sufficiently large sample to train models that identify patients with IDHwt GBM. These documents were manually assessed for tumours that aligned with the 2021 WHO definition. In parallel with manual review, all notes from the NMEDW containing the same text string were processed using the National Library of Medicine's MetaMap tool [27] to extract all Unified Medical Language System (UMLS) concepts contained in the text [28]. These concepts were used as covariates, and our manually assigned GBM labels were used as the response variables to train penalized logistic regression models to recognize IDHwt GBM. The best performing model in the remaining 20% of notes was then implemented on all pathology documents in the NMEDW containing the text string to isolate our preliminary cohort (F‐measure 0.88). Patients were retained in the analytic cohort based on the same weight and BMI data availability criteria specified in Section 2.2.

#### 2.3.1 Study Measures

##### 2.3.1.1 Baseline Consensus Criteria

We extracted each patient's maximum weight during the 6 months prior to initial cancer diagnosis. This maximum weight was subtracted from the weight measurement that occurred closest in time relative to the initial cancer diagnosis. This difference was divided by the maximum weight to obtain the percent weight change at baseline. Among patients with a BMI ≥ 20, > 5% weight loss was labelled as ‘cachexia’, 1%–5% as ‘pre‐cachexia’, and less than 1% as ‘no cachexia’. In patients with a BMI < 20, > 2% weight loss was labelled as ‘cachexia’. Outliers that either exceeded the 75th percentile of all baseline measurements added to 1.5 times the interquartile range or fell below 1.5 times the interquartile range subtracted from the 25th percentile were removed [29]. A parallel analysis using the Weight Loss Grading Scale (WLGS) was also conducted (see Supporting Information) [6].

##### 2.3.1.2 Clinical Endpoints

Survival analyses were conducted using three endpoints occurring after the initial cancer diagnosis to analyse the association between cachexia severity and mortality, functional status or morbidity. Overall survival was defined as time to death by any cause. When no death date was available, patients were censored using the last available clinical encounter date. Disability‐free survival was defined as the time to the first order for rehabilitation services following cancer diagnosis. Patients in this analysis were censored if death occurred prior to order of rehabilitation services or at the time of the last clinical encounter. Patients who received a rehabilitation order prior to the date of cancer diagnosis were excluded from this analysis. Hospitalization‐free survival was defined as the time to the first inpatient clinical encounter following cancer diagnosis.

#### 2.3.2 Statistical Analysis

All analyses were performed in the R software environment, Version 4.1.2.

##### 2.3.2.1 Descriptive Statistics

Variables pertaining to race, sex, ethnicity and age at cancer diagnosis were extracted from the NMEDW. Measures of central tendency and spread were computed for continuous variables and frequency for categorical variables. Variables pertaining to functional status—Eastern Cooperative Oncology Group (ECOG) for lung cancer [30] and Karnofsky Performance Status (KPS) for GBM [31]—were extracted from the NMEDW using both structured data and narrative documents. Baseline values for these variables were obtained by isolating the measurement that occurred closest to initial cancer diagnosis. KPS was extracted among patients in the GBM cohort based on its ubiquitous use in the setting of central nervous system malignancies [32]. Measurements occurring greater than 6 months prior to or following initial diagnosis were excluded. Cancer stage was obtained in a similar manner only for patients with lung cancer. Disease severity in GBM was measured using anatomic indicators of invasiveness, identified through manual adjudication of radiology and neuro‐oncology documents in the EHR. O(6)‐Methylguanine‐DNA methyltransferase (MGMT) promoter methylation status, a molecular marker of GBM chemosensitivity [33], was extracted using a model analogous to that used in identification of the GBM cohort, described in Section 2.1. Finally, the number of weight measures following cancer diagnosis and the number of weight measures divided by the length of follow‐up were computed to assess data availability and density. A summary of all text searches utilized in identifying these variables is summarized in Table S1.

##### 2.3.2.2 Consensus Criteria Trajectory Identification

To identify temporal trends of consensus criteria measures, we first extracted all weight and BMI measurements that occurred on or after the date of a patient's cancer diagnosis. Each measurement was converted to one of the three consensus criteria categories that were then assigned an ordinal value in order of increasing cachexia severity, resulting in a numeric sequence of consensus criteria measurements for each patient. The similarity between patients' sequences of cachexia measures was calculated via DTW. DTW first calculates a pairwise distance between each combination of the sequences' elements arranged in chronological order by a user‐defined distance function [34]. The pairwise distance in this study is the difference between the cachexia severity ordinal values. The algorithm then walks iteratively through the pairwise distance matrix, choosing a path that minimizes the cost of aligning points between the sequences. The cost of each walk step is defined as the distance in the current matrix cell summed with the minimum cost of three previous cells in either the lateral, diagonal or vertical directions. The cumulative cost resulting at the end of the iterative walk is equal to the DTW similarity. All DTW similarity values were normalized by the sum of the length of the two sequences being compared to minimize dependency on the sequence length [34]. The DTW similarity between patient sequences was passed to a K‐medoids clustering algorithm that sorted patients into subgroups [35]. The optimum number of subgroups, referred to as clusters, was determined by finding the maximum average silhouette width across *k* number of clusters equal to two through ten. We also computed a null clustering solution that used the difference between the numbers of weight measurements per patient as the distance metric. We subsequently computed the Adjusted Rand Index (ARI) between temporal clusters and the null clusters in each cohort, which assumes values of zero between two random clustering algorithms and a value of one for perfect agreement [36]. A demonstration of our DTW implementation and subsequent clustering has been made available at https://github.com/noahnwu/DynamicTimeWarping.

##### 2.3.2.3 Trajectory Characterization and Cluster Comparison

The percentage of patients with cachexia—as defined by the consensus criteria—within each cluster was computed at 1‐week intervals by dividing the number of patients with cachexia by the number of patients still alive and not lost to follow‐up at each time point. The same set of descriptive statistics extracted for the overall cohorts were further subdivided and summarized by patient cluster membership. Within‐cohort clusters were then compared quantitatively to identify differences among patients assigned to each cluster. Nominal variables were compared via chi‐square tests, continuous and normally distributed variables by one‐way analysis of variance and ordinal or non‐normally distributed variables using the Kruskal–Wallis one‐way ANOVA test. All statistical tests described were conducted at the 95% confidence level.

##### 2.3.2.4 Survival Analysis

Kaplan–Meier curves were visualized using the *survival* package in R, Version 3.2‐13 [37]. Strata were compared using the log‐rank test.

##### 2.3.2.5 Predictive Capacity

To compare the ability of baseline and temporal trajectories to stratify outcomes, we employed an inverse probability of censoring weighting (IPCW) [38] estimation of time‐dependent receiver operating characteristic curves (ROC) to compute the areas under the curve (AUCs) at 1‐month intervals starting from 1 month after diagnosis until the median overall survival time was reached for each cohort. Calculation of the AUC, interpreted analogously to the concordance index, requires that the predictor be dichotomized into cases and controls. For the baseline measurement, cases were defined as patients with cachexia. Cases were defined as patients categorized into the ‘persistent or recurrent’ cluster in the temporal trajectory analysis. We also constructed univariate and multivariate Cox proportional hazards models using either the baseline measurement or temporal trajectory to compute overall concordance. All variables were dichotomized to limit model complexity. Multivariate models utilized one of the consensus criteria‐related variables in combination with sex, race, ethnic group, functional status as measured by either KPS or ECOG, cancer‐specific severity markers and age. We also constructed multivariate Cox models without race and ethnicity as a sensitivity test on their impact on cachexia‐related coefficients.

## 3 Results

### 3.1 Cohort Attributes

**FIGURE 1:**
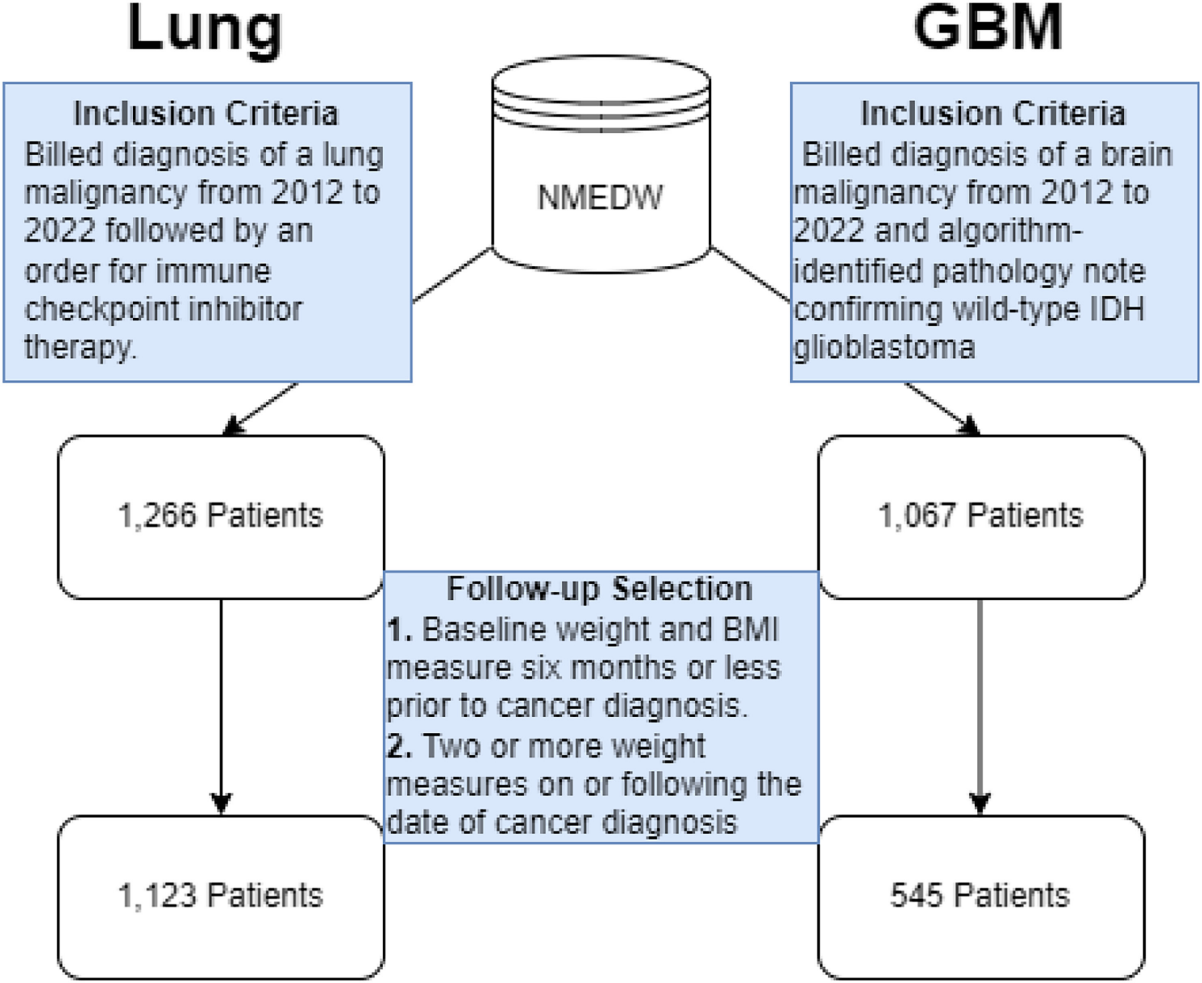
Patient inclusion diagram. Left: Patients with lung cancer were initially included if a billed diagnosis of a lung malignancy was documented between 1 January 2012 and 31 December 2022 and if an order for administration of an immune checkpoint inhibitor occurred following the diagnosis. Right: Patients with GBM were identified by first querying the EDW for all patients with a billed diagnosis of a brain malignancy. Pathology documents were extracted from these patients that contained the word ‘glioblastoma’. A subset of these documents were reviewed for the presence of wild‐type IDH glioblastoma and a supervised machine learning algorithm was trained to identify patients matching this phenotype. Center: In both cohorts, patients were retained for analysis if the medical record contained a baseline weight measurement, defined as any measure made 6 months or less prior to diagnosis. To conduct the longitudinal analysis, patients were also required to have two or more weight measures following cancer diagnosis.

We identified 1123 patients with lung cancer and 545 patients with GBM matching our inclusion criteria, described in Figure 1. Descriptive baseline statistics obtained from patients in both cohorts are summarized in Table 1. The majority of patients in the lung cancer cohort were Stage IV (53%) at diagnosis, displayed a high performance status (79% ECOG  1) and exhibited consensus‐criteria defined pre‐cachexia or cachexia at baseline (60%). The 1‐ and 5‐year overall survival probabilities among lung cancer patients were 0.75 (0.73–0.78) and 0.36 (0.32–0.40), respectively, with 592 events identified. Among patients with GBM, nearly half of individuals displayed a high performance status (KPS 80–100), 82% of patients possessed unilateral cortical tumours and 51% displayed consensus criteria‐defined cachexia at baseline. A median of 48 (IQR = 55) weight measures following diagnosis among patients with lung cancer and 18 (IQR = 23) among those with GBM. Patients with lung cancer also displayed a high density of weight measurements, with a median weight measures per year of 31 (IQR = 28). Patients with GBM displayed a median of 17 weight measures per year (IQR = 15). The 1‐ and 5‐year overall survival probabilities among patients with GBM were 0.65 (0.61–0.70) and 0.11 (0.08–0.16), respectively, with 353 events detected.

**TABLE 1:** Descriptive statistics of lung cancer and glioblastoma cohorts.

Variable category	Variable	Lung			Glioblastoma		
		*N* = 1123			*N* = 545		
Demographic information	**Age at diagnosis**	**Mean (SD)**	**Median [min, max]**		**Mean (SD)**	**Median [min, max]**	
		68 (10)	68 [23, 89]		61 (13)	63 [18, 89]	
	**Race**	***N***	**%**		***N***	**%**	
	White	881	78		437	80	
	Black or African American	127	11		19	3	
	Other	115	10		89	16	
	**Sex**						
	M	562	50		316	58	
	F	561	50		229	42	
	**Ethnicity**						
	Not Hispanic or Latino	1088	97		514	94	
	Hispanic or Latino	35	3		31	6	
Functional status	**Baseline ECOG**						
	0	369	33		—	—	
	1	514	46		—	—	
	2	144	13		—	—	
	3	28	2		—	—	
	4	1	< 1		—	—	
	Unavailable	67	6		—	—	
	**Baseline KPS**						
	0–30	—	—		13	2	
	31–60	—	—		54	10	
	61–80	—	—		186	34	
	80–100	—	—		264	48	
	Unavailable	—	—		28	5	
Cancer severity	**Cancer stage**						
	I	91	8		0	0	
	II	57	5		0	0	
	III	264	24		0	0	
	IV	590	53		545	100	
	Unavailable	121	11		0	0	
	**Primary tumour site**						
	Unilateral cortical	—	—		447	82	
	Bi‐hemispheric cortical	—	—		26	4	
	Non‐cortical or multifocal	—	—		72	13	
	**MGMT promoter**						
	Methylated	—	—		221	41	
	Unmethylated	—	—		305	56	
	Unknown	—	—		19	3	
Baseline cachexia severity	**Baseline consensus category**						
	No cachexia	453	40		136	25	
	Pre‐cachexia	399	36		130	23	
	Cachexia	271	24		279	51	
Time to event, follow‐up and survival	**Survival years**	**N events** ^a^	**Median** ^**b**^	**95% CI**	**N events**	**Median**	**95% CI**
	Overall	592	2.9	2.6–3.2	353	1.3	1.2–1.5
	Disability‐free	483	3.1	2.7–3.7	294	0.4	0.1–0.8
	Hospitalization‐free	976	0.3	0.3–0.4	381	0.5	0.4–0.7
	**Years of follow‐up** ^**c**^	**Median**	**[Min, max]**		**Median**	**[Min, max]**	
		1.7	[0.01, 10]		1.03	[0.03, 8.45]	
		**Median**	**IQR** ^**d**^		**Median**	**IQR**	
	**N weight measures** ^**e**^	48	55		18	23	
	**Weight measures/follow‐up** ^**f**^	31	28		17	15	
	**Survival probability (95% CI)** ^**g**^	**One‐year**	**Five‐year**		**One‐year**	**Five‐year**	
	Overall	0.75 (0.73–0.78)	0.36 (0.32–0.40)		0.65 (0.61–0.70)	0.11 (0.08–0.16)	
	Disability‐free	0.72 (0.69–0.75)	0.36 (0.31–0.40)		0.43 (0.39–0.48)	0.15 (0.10–0.24)	
	Hospitalization‐free	0.27 (0.24–0.29)	0.06 (0.05–0.08)		0.34 (0.30–0.39)	0.1 (0.06–0.16)	

*Note:* Summary statistics were obtained for each cohort using a combination of structured data and text searches in patients' electronic health records.

Abbreviations: ECOG, Eastern Cooperative Oncology Group; KPS, Karnofsky Performance Status Scale; MGMT, O‐6‐methylguanine‐DNA methyltransferase.

^a^ Number of events observed for each of the three outcomes studied.

^b^ Median survival time for each of the three outcomes studied, in years following initial cancer diagnosis.

^c^ Median time from initial diagnosis to death or loss to follow‐up.

^d^ Interquartile range.

^e^ Total number of weight measures detected per patient following initial cancer diagnosis.

^f^ Total number of measures divided by the total length of follow‐up.

^g^ Survival probability computed from Kaplan–Meier curves fit in R software environment.

### 3.2 Baseline Measurements of Cachexia Stratify Outcomes in Lung Cancer but Not in GBM

**FIGURE 2:**
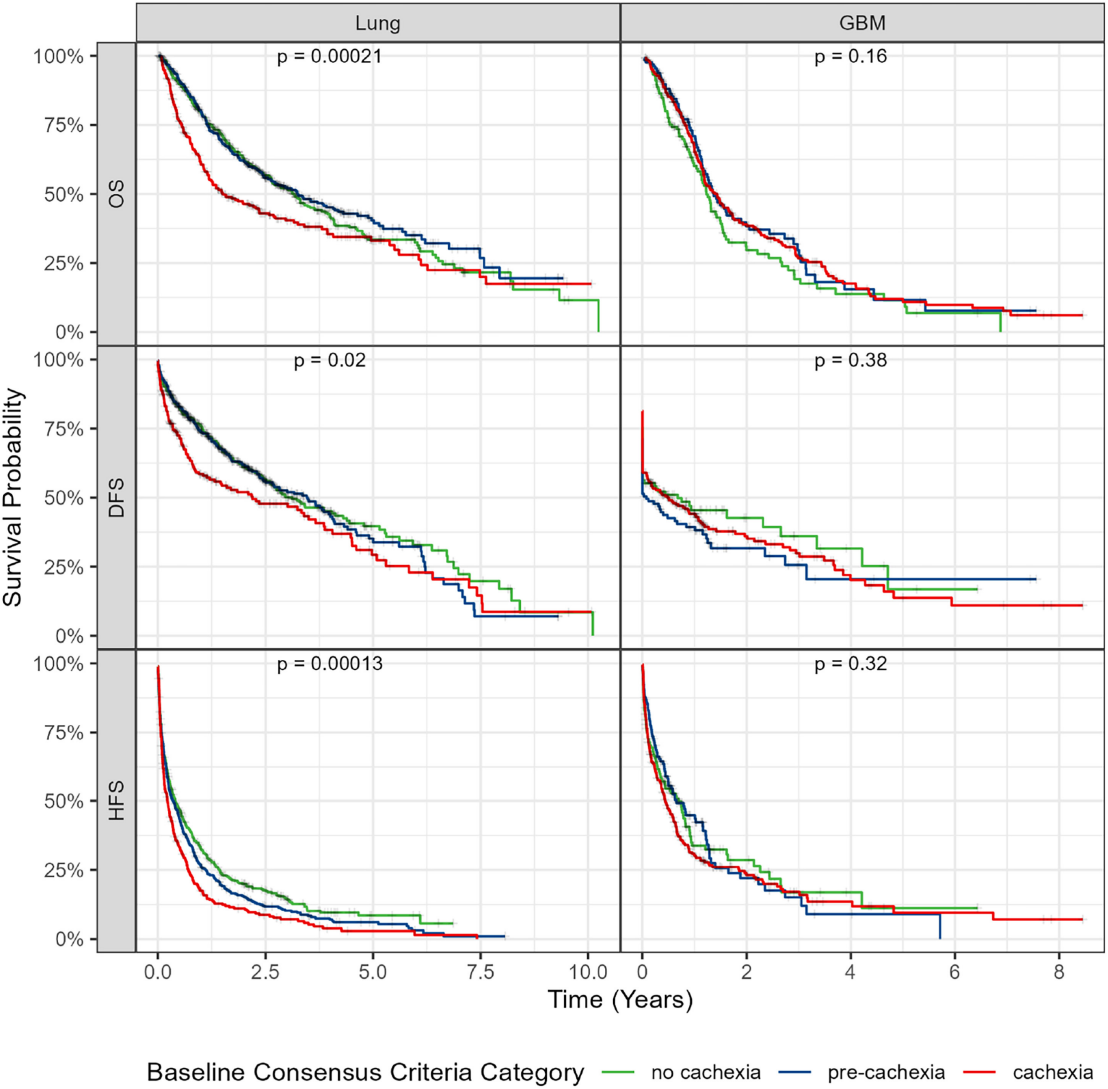
Baseline measurements of cachexia stratify outcomes in lung cancer but not in GBM. Kaplan–Meier curves were constructed, stratifying patients by their baseline measure. *p* values displayed in each panel were computed using the log‐rank test. DFS, disability‐free survival; HFS, hospitalization‐free survival; OS, overall survival.

Figure 2 shows the Kaplan–Meier curves for overall, disability‐free and hospitalization‐free survival when patients were stratified by baseline cachexia status. The baseline consensus criteria category successfully stratified survival among patients with lung cancer, with *p* values of < 0.001, 0.0018 and < 0.001 for overall, disability‐free and hospitalization‐free survival., respectively. No significant differences in the three survival curves were observed in the GBM cohort, with *p* values of 0.16, 0.38 and 0.32 for overall, disability‐free and hospitalization‐free survival., respectively. These findings suggest that a single baseline measurement of cachexia may only be prognostically useful in some cancer contexts and may not apply to others, such as GBM, consistent with previous work [9]. Similar results were observed when stratifying patients by their baseline WLGS measure (Figure S1).

### 3.3 DTW Identifies Patterns of Cachexia Progression

**FIGURE 3:**
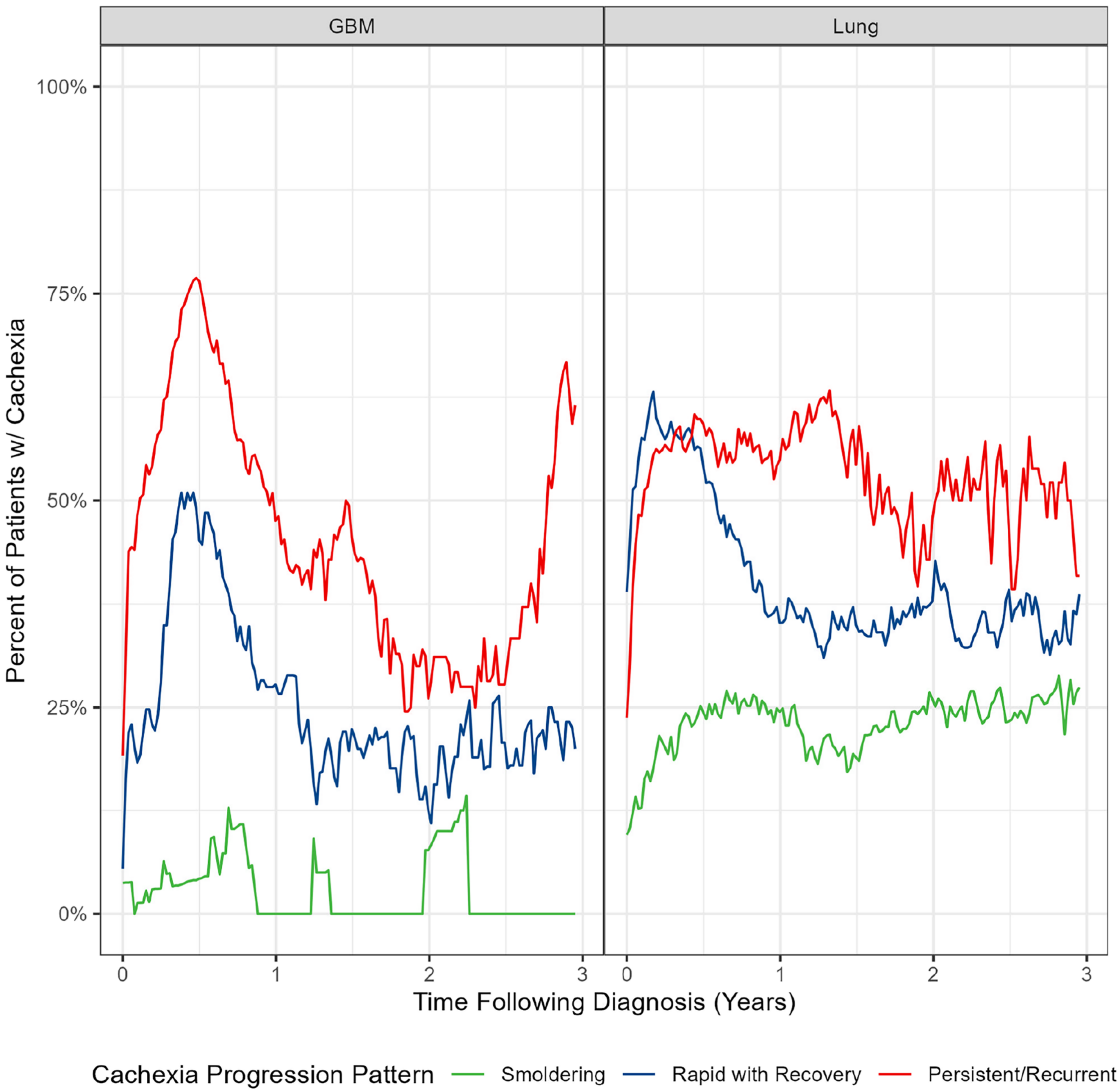
Dynamic time warping and clustering on longitudinal consensus criteria measurements identifies patterns of cachexia progression. The percentage of patients still alive with criteria‐defined cachexia within each cluster was computed at 1‐week intervals from the time of cancer diagnosis for the following 3 years to illustrate the subgroup cachexia progression patterns. GBM, glioblastoma; Lung, lung cancer.

We next sought to develop a system for categorizing patients based on longitudinal patterns of cachexia severity. Our analysis revealed three sub‐populations in both cohorts. Figure 3 displays the percentage of patients who were alive and not lost to follow‐up with consensus criteria‐defined cachexia among each subgroup. Among patients with lung cancer, Group 1 (red) consisted of patients where the frequency of cachexia was relatively high on the date of cancer diagnosis and remained high following initial cancer diagnosis. Group 2 (green) consisted of patients in which the frequency of cachexia was relatively low on the date of initial diagnosis and remained low. Finally, Group 3 (blue) was made up of patients where the frequency of cachexia on the date of diagnosis was high, increased to a large proportion and quickly decreased. Similar patterns were observed among the subgroups identified in the cohort of patients with GBM. These findings suggest that distinct temporal progression patterns of cachexia can be uncovered in populations of patients with cancer. Clusters 1, 2 and 3 are referred to as ‘persistent or recurrent’, ‘smouldering’ and ‘rapid with recovery’ progression patterns, respectively. An additional visualization highlighting the rate of cachexia progression in the three patient subgroups is shown in Figure S2, which displays Kaplan–Meier curves for ‘cachexia‐free’ survival. We also observed distinct patterns of progression when using longitudinal WLGS measures as inputs into the DTW–clustering pipeline (Figure S3). In comparison to null clustering solutions, the ARIs were 0.18 and 0.11 for Lung and GBM, respectively, indicating a low agreement between the null models and the temporal patterns described and suggesting that cluster assignment was not influenced by the number of measures observed for each patient.

Table 2 summarizes demographic and clinical information of patients stratified by their cachexia progression pattern. Among patients with lung cancer, significant differences were observed between subgroups for cancer stage, baseline ECOG, density of weight measures and the baseline consensus criteria category, with *p* values of < 0.001 observed for each variable. The subgroup with the ‘persistent’ progression pattern displayed the highest percentage of patients with Stage IV disease (60%). Individuals with the smouldering progression displayed the highest baseline functional status, with 43% of this group having an ECOG score of 0. Finally, the group with a ‘rapid with recovery’ cachexia progression pattern displayed the highest percentage of individuals with cachexia at baseline (35%). Significant differences were observed between temporal progression subgroups in the GBM cohort for the age at diagnosis, baseline KPS, primary tumour site, density of weight measures and the baseline consensus criteria cachexia category, with *p* values of < 0.001, < 0.001, 0.04, < 0.001 and 0.002, respectively. Individuals categorized within the ‘rapid with recovery’ subgroup were generally younger, with a mean age at diagnosis of 56 years, compared to the ‘persistent or recurrent’ and ‘smouldering’ groups. Patients with the rapid with recovery pattern also displayed the highest proportion of individuals at the highest KPS functional status category (KPS > 80), making up 66% of this subgroup. This group displayed the highest percentage of patients with consensus criteria‐defined cachexia at baseline. Finally, patients in the persistent or recurrent group displayed the highest rates of tumours located in non‐cortical brain areas and bi‐hemispheric tumours, indicative of more advanced disease. Taken together, these findings indicate that baseline features of functional status, age and tumour‐specific features of disease severity are associated with distinct patterns of cachexia progression.

**TABLE 2:** Clinical characteristics differ between patients with distinct cachexia progression patterns.

Variable category	Temporal cachexia pattern	Lung			GBM		
		Persistent	Smouldering	Rapid w/ recovery	Recurrent	Smouldering	Rapid w/ recovery
	Variable	*N* = 429	*N* = 263	*N* = 431	*N* = 355	*N* = 80	*N* = 110
Demographic information	**Race: *N* (%)**			***p* = 0.45** ^**a**^			***p* = 0.50**
	White	329 (77)	206 (78)	346 (80)	278 (78)	65 (81)	94 (85)
	Black or African American	48 (11)	34 (13)	45 (10)	15 (4)	2 (2)	2 (2)
	Other	52 (12)	23 (9)	40 (9)	62 (17)	13 (16)	14 (13)
	**Sex: *N* (%)**			***p* = 0.22**			***p* = 0.23**
	M	228 (53)	123 (47)	211 (49)	215 (61)	44 (55)	57 (52)
	F	201 (47)	140 (53)	220 (51)	140 (39)	36 (45)	53 (48)
	**Ethnicity: *N* (%)**			***p* = 0.40**			***p* = 0.06**
	Not Hispanic or Latino	413 (96)	258 (98)	417 (97)	337 (95)	78 (98)	99 (90)
	Hispanic or Latino	16 (4)	5 (2)	14 (3)	18 (5)	2 (2)	11 (10)
	**Age at diagnosis in years**			***p* = 0.10**			***p* < 0.001**
	Mean (SD)	69 (10)	67 (9)	67 (10)	62 (13)	65 (13)	56 (13)
	Median [min, max]	69 [30, 89]	68 [32, 88]	68 [22, 89]	64 [18, 89]	66 [25, 88]	57 [21, 86]
Functional status	**Baseline ECOG: *N* (%)**			***p* < 0.001**			—
	0	115 (27)	112 (43)	142 (33)	—	—	—
	1	197 (46)	110 (42)	207 (48)	—	—	—
	2	69 (16)	25 (10)	50 (12)	—	—	—
	3	14 (3)	5 (2)	9 (2)	—	—	—
	4	1 (<1)	0	0	—	—	—
	Unavailable	33 (8)	11 (4)	23 (5)	—	—	—
	**Baseline KPS: *N* (%)**			—			***p* < 0.001**
	0–30	—	—	—	2 (1)	0	0
	31–60	—	—	—	45 (13)	5 (6)	4 (4)
	61–80	—	—	—	128 (36)	25 (31)	25 (23)
	81–100	—	—	—	138 (39)	38 (48)	73 (66)
	Unavailable	—	—	—	42 (12)	12 (15)	8 (7)
Cancer severity	**Cancer stage: *N* (%)**			***p* < 0.001**			—
	I	18 (4)	42 (16)	31 (7)	—	—	—
	II	14 (3)	18 (7)	25 (6)	—	—	—
	III	75 (17)	68 (26)	121 (28)	—	—	—
	IV	259 (60)	115 (44)	216 (50)	355 (100)	80 (100)	110 (100)
	Unavailable	63 (15)	39 (12)	38 (9)	—	—	—
	**Primary tumour site**			—			***p* = 0.04**
	Unilateral cortical			—	281 (79)	73 (91)	93 (85)
	Bi‐hemispheric cortical	—	—		23 (6)	0 (0)	3 (2)
	Non‐cortical or multifocal	—	—		51 (14)	7 (9)	14 (13)
	**MGMT promoter status: *N* (%)**			—			***p* = 0.05**
	Methylated	—	—	—	144 (40)	35 (44)	42 (38)
	Unmethylated	—	—	—	203 (57)	43 (54)	59 (54)
	Unknown	—	—	—	8 (2)	2 (3)	9 (8)
Baseline cachexia severity	**Baseline consensus category**			***p* < 0.001**			***p* = 0.002**
	No cachexia	189 (44)	132 (50)	132 (31)	101 (28)	21 (26)	14 (13)
	Pre‐cachexia	144 (34)	106 (40)	149 (35)	81 (23)	25 (31)	24 (22)
	Cachexia	96 (22)	25 (10)	150 (35)	173 (49)	34 (42)	72 (65)
Weight measures	**Mean N measures (SD)**			***p* < 0.001**			***p* < 0.001**
		31 (21)	93 (53)	67 (37)	20 (15)	9 (7)	45 (28)
	**Mean N measures/follow‐up (SD)**			***p* < 0.001**			***p* < 0.001**
		53 (42)	30 (20)	37 (26)	24 (24)	15 (11)	20 (12)
Time to event and survival	**Median year survival (95% CI)**						
	Overall	0.9 (0.8–1.1)	6.1 (5.7–7.6)	3.5 (2.9–4.1)	1.1 (1.0–1.2)	1.1 (0.9–2.2)	3.4 (2.7–4.4)
	Disability‐free	1.2 (1.0–1.5)	4.8 (4.1–5.9)	3.0 (3.4–3.9)	0.1 (0–0.5)	0.7 (0–NA)	2.3 (0.9–3.7)
	Hospitalization‐free	0.3 (0.2–0.3)	0.5 (0.3–0.8)	0.3 (0.2–0.4)	0.4 (0.3–0.6)	1.2 (0.6–NA)	0.7 (0.4–0.9)
	**One‐year survival** ^**b**^ **(95% CI)**						
	Overall	0.49 (0.44–0.54)	0.94 (0.91–0.97)	0.87 (0.84–0.90)	0.58 (0.54–0.64)	0.53 (0.42–0.68)	0.92 (0.87–0.98)
	Disability‐free	0.57 (0.52–0.64)	0.86 (0.82–0.90)	0.73 (0.68–0.77)	0.37 (0.32–0.44)	0.48 (0.38–0.61)	0.58 (0.49–0.69)
	Hospitalization‐free	0.19 (0.15–0.23)	0.38 (0.33–0.45)	0.26 (0.23–0.31)	0.31 (0.26–0.37)	0.52 (0.41–0.67)	0.36 (0.28–0.47)
	**Five‐year survival (95% CI)**						
	Overall	0.08 (0.04–0.15)	0.59 (0.52–0.67)	0.41 (0.35–0.47)	0.02 (0.02–0.11)	0.25 (0.15–0.46)	0.25 (0.17–0.39)
	Disability‐free	0.23 (0.14–0.37)	0.48 (0.41–0.57)	0.34 (0.28–0.42)	0.20 (0.13–0.30)	0.21 (0.05–0.85)	0.19 (0.10–0.34)
	Hospitalization‐free	0.04 (0.02–0.08)	0.12 (0.08–0.17)	0.05 (0.03–0.09)	0.07 (0.03–0.18)	0.17 (0.04–0.73)	0.12 (0.06–0.25)

*Note:* Summary statistics were obtained for each cluster discovered in the two cohorts. Statistical comparisons between clusters were conducted within each cohort utilizing chi‐square for categorical variables and one‐way ANOVA for continuous variables or its non‐parametric equivalent to assess for differences in baseline clinical characteristics between patients with differing cachexia progression patterns.

^a^ Reported *p* values reflect whether significant differences were detected between same‐cohort cachexia progression subgroups at the 95% confidence level. *p* values for time‐to‐event analyses reported in corresponding figures.

^b^ One‐ and five‐year survival probabilities computed from Kaplan–Meier curves constructed in the R software environment.

### 3.4 Cachexia Progression Patterns Stratify Outcomes in Lung Cancer and GBM

**FIGURE 4:**
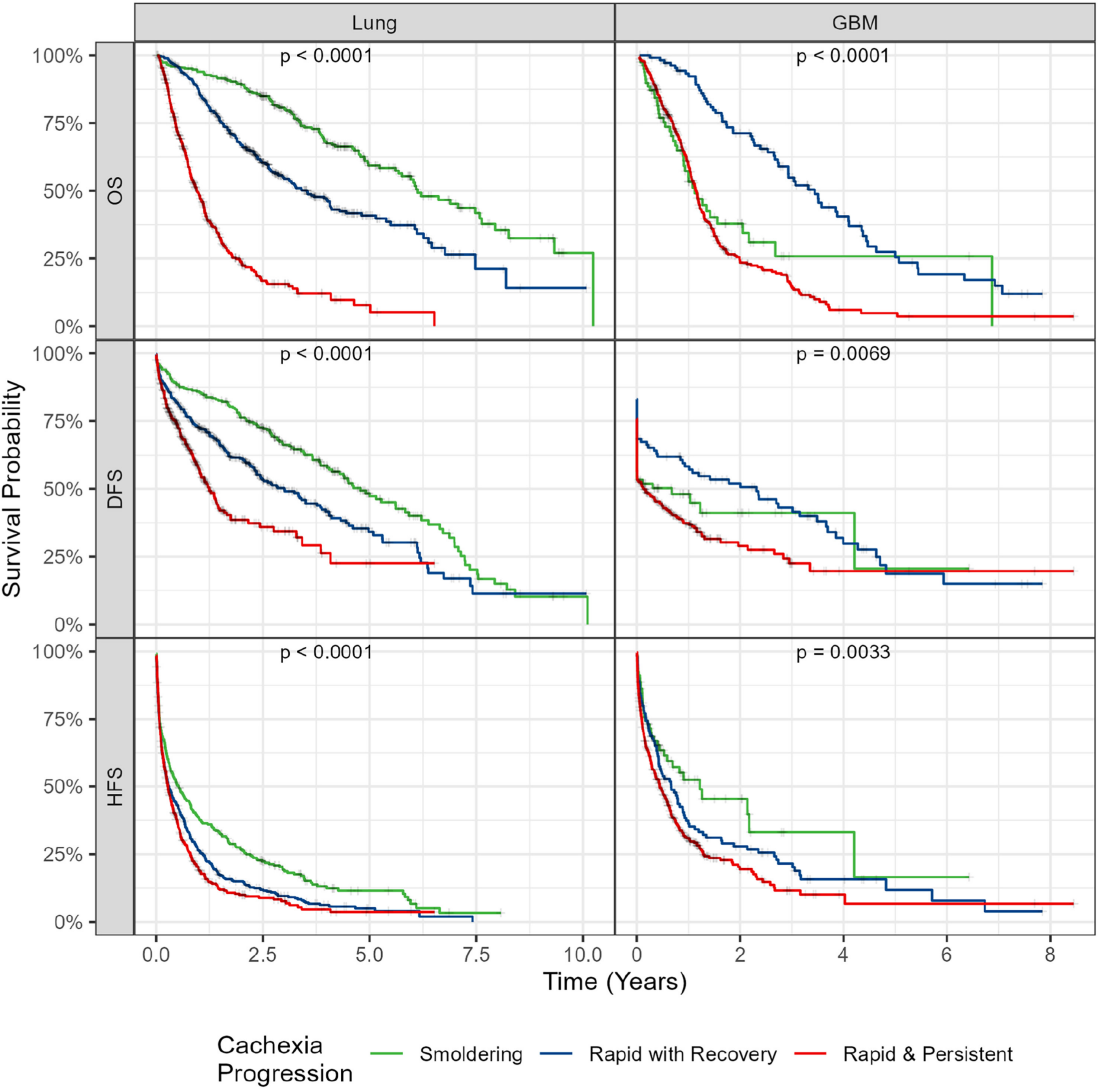
Cachexia progression patterns stratify patient outcomes in lung cancer and GBM. Kaplan–Meier curves of three patient‐centred outcomes among patients with lung cancer and GBM, stratified by their temporal cachexia progression pattern. *p* values were computed using the log‐rank test. DFS, disability‐free survival; HFS, hospitalization‐free survival; OS, overall survival.

Figure 4 displays the Kaplan–Meier curves stratified by temporal cluster for overall, disability‐free and hospitalization‐free survival. Significant differences were observed between Kaplan–Meier curves of the cachexia progression groups for all outcomes studied in patients with lung cancer, with *p* values of < 0.001. The smouldering progressing group displayed the longest time to each outcome followed by those with a ‘rapid with recovery’ progression pattern. Those with a persistent progression pattern displayed the least favourable survival curve for all outcomes. Among patients with GBM, significant differences between Kaplan–Meier curves of the cachexia progression groups were observed for all outcomes, with *p* values of < 0.001, 0.0069 and 0.0033 for overall, disability‐free and hospitalization‐free survival, respectively. The ‘rapid with recovery’ progression group typically displayed the longest time to reach each outcome, except for hospitalization‐free survival, for which the smouldering progressing group displayed the most favourable survival curve. Patients with a persistent/recurrent progression pattern displayed the poorest outcome curves for all outcomes studied. A post hoc analysis comparing each pair of clusters is shown in Table S2. From these results, we conclude that temporal progression patterns of cachexia successfully stratify patient outcomes. When stratifying patients by their WLGS‐based progression pattern, significant differences in survival were also observed between strata for two of the three outcomes studied among patients with lung cancer. We were unable to observe significant differences in outcomes among patients with GBM (Figure S4).

### 3.5 Cachexia Progression Patterns Better Stratify Patient Outcomes at Multiple Time Points

**FIGURE 5:**
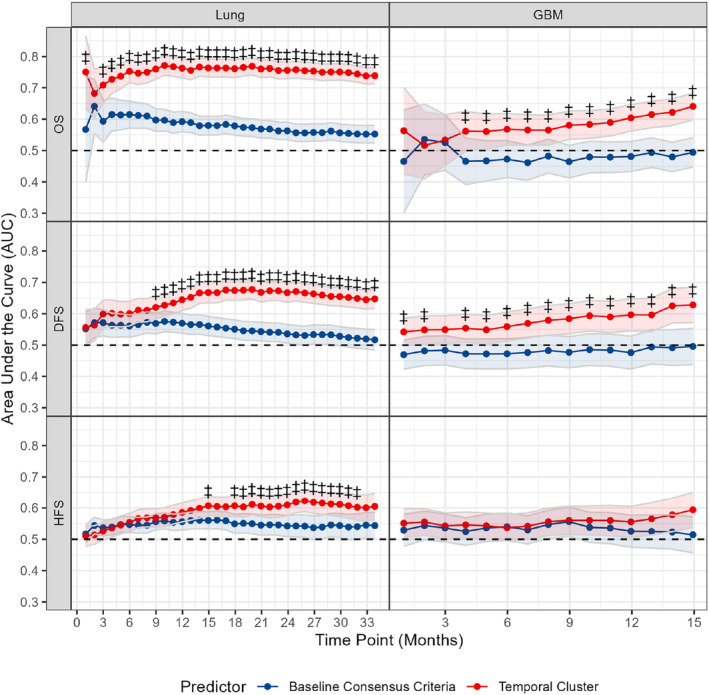
Cachexia progression patterns stratify patient outcomes more effectively than baseline measures at multiple time points. Areas under the curve (AUC) for the baseline cachexia measure and temporal cachexia patterns were computed at 1‐month intervals starting at the time of cancer diagnosis until the median overall survival time was reached in each cohort. Values were compared between the baseline 2011 consensus criteria measure and temporal clusters to identify differences in cachexia measure performance in stratifying patient outcomes. The AUC describes the frequency with which the higher risk group—defined as cachexia for the baseline measurement and ‘persistent or recurrent’ for the temporal clusters—predict a shorter time to event than lower risk groups. Higher AUC values indicate variables that more effectively stratify patient outcomes. DFS, disability‐free survival; HFS, hospitalization‐free survival, OS; overall survival. Shaded region: confidence interval at the 95% confidence level. ‡Significant difference between receiver operating curves of baseline and temporal cluster Kaplan–Meier predictions at indicated time point and 95% confidence level.

Figure 5 displays the time‐dependent AUCs computed for the baseline consensus criteria measurement and temporal pattern subgroups. Among patients with lung cancer, significant differences between AUCs were observed at multiple time points for all outcomes examined, with the temporal clusters generally displaying significantly higher AUC values. Similar patterns were observed when comparing the two consensus criteria variations in patients with GBM. WLGS‐based temporal groups also displayed higher AUC values than single WLGS baseline measures at multiple time points following diagnosis in patients with lung cancer though not in GBM (Figure S5). The overall concordance indices from fitting Cox proportional hazards models for each outcome are outlined in Table 3. The concordance index was generally higher when using the temporal cluster as a covariate as opposed to the baseline measurement in multivariate models, except for hospitalization‐free survival in lung cancer. The difference in concordance between models incorporating baseline measures or temporal cluster membership was especially pronounced with multivariate adjustment for overall survival in the lung cancer cohort (0.636 vs. 0.735). These findings suggest that the temporal progression patterns generally perform better in prognosticating patient outcomes than single baseline measures. The hazards ratios (HR) of Cox models comparing the ‘persistent/recurrent’ subgroup versus all other individuals were 4.8 (4.1–5.8), 2.0 (1.7–2.5) and 1.3 (1.1–1.4) fit to overall, disability‐free and hospitalization‐free survival, respectively, following adjustment for covariates. Analogous Cox models in the GBM cohort displayed HR of 1.9 (1.4–2.4), 1.3 (1.01–1.7) and 1.3 (1.0–1.7) following adjustment. These results were not dramatically altered with the removal of race and ethnicity from the models. HR estimates of all covariates resulting from univariate models, multivariate models with race and ethnicity and multivariate models without race and ethnicity are detailed in Tables S3–S8.

**TABLE 3:** Cachexia progression patterns stratify patient outcomes more effectively than baseline measures overall.

	Outcome	Univariate^a^		Full model^b^	
		Baseline	Temporal pattern	Baseline	Temporal pattern
Lung	OS	0.554	0.680	0.636	0.735
	DFS	0.539	0.579	0.595	0.619
	HFS	0.524	0.521	0.555	0.544
GBM	OS	0.513	0.585	0.684	0.693
	DFS	0.522	0.542	0.579	0.581
	HFS	0.527	0.541	0.610	0.614

*Note:* Concordance statistics obtained from Cox proportional hazards models constructed using either baseline measures or cachexia progression patterns as strata. The concordance statistic describes the degree of agreement between a predictor variable(s) and an outcome, with higher values indicating a better model fit.

Abbreviations: DFS, disability‐free survival; HFS, hospitalization‐free survival; OS, overall survival.

^a^ Cox proportional hazards model containing only the consensus criteria measure of interest.

^b^ Cox model containing either baseline consensus category or temporal cachexia progression pattern adjusted for age, ethnicity, race, functional status (ECOG in lung cancer and KPS in GBM), MGMT promoter status (GBM only), cancer stage (lung cancer only), tumour location (GBM only) and sex.

## 4 Discussion

Our goal was to develop a temporal extension to the Fearon consensus criteria and compare it against a single measure at baseline in stratifying three patient‐centred outcomes. Stratification by the baseline consensus measure did show significant differences in survival curves in lung cancer but not in GBM. This highlights that single time point measures of the consensus criteria may only have value in cancer‐specific contexts. We then conducted the same analysis stratifying patients by three temporal clusters identified by DTW and clustering and observed significant differences between curves for all outcomes studied in both cancer cohorts. Finally, we assessed the predictive capability of our algorithm against that of the baseline measurement, which showed improved metrics of fit at multiple time points. These results suggest that accounting for the longitudinal progression of cachexia may be valuable for improving cachexia detection and prognosis in cancer contexts for which the impact of cachexia is not well recognized, such as GBM.

We also observed differences in baseline clinical variables between patients identified with each cachexia progression pattern. Lung cancer patients in the ‘persistent’ progression subgroup displayed relatively poor functional status measured by ECOG and the highest percentage of patients with Stage IV disease. Patients with GBM in the smouldering progression group did not have the longest time to progression for all outcomes as was observed in lung cancer. There may be several confounding reasons that account for this paradoxical outcome stratification. First, patients in this group displayed the highest age and poorest functional status at diagnosis, on average, compared to the other two progression pattern groups, suggesting that there may have been a higher degree of pre‐existing sarcopenia. Advanced age and poor functional status often warrant reduced chemotherapeutic regimens and hypofractionation of radiotherapy in GBM [39]. Indeed, we did note a higher proportion of patients without evidence of radiotherapy and the standard chemotherapeutic regimen among patients in the subgroup of patients with the ‘smouldering’ phenotype (Table S9). Overall, our findings in these two cohorts suggest that the temporal progression patterns may be influenced by other clinical factors.

There are several strengths to the systems developed in this work. Our analyses circumvent the need to extract a measurement from the EHR at an arbitrarily defined time point and can use any number of measures following cancer diagnosis, as the DTW algorithm only accounts for shapes of longitudinal trajectories and ignores the length of the sequence or when the measurement occurred. This is supported by the fact that our null clustering model based on sequence length did not agree with the temporal subgroups described. To define our GBM cohort, many patients were excluded due to the lack of a baseline weight measurement, which we defined as the measurement at any point 6 months prior to the initial cancer diagnosis. Our system would allow inclusion of patients with such missing data. Finally, the patterns of weight loss progression within each cluster are easily interpretable, making it a potentially useful clinical tool in the future.

Certain limitations are present due to the nature of EHR data, available resources and the tumour types included in this proof‐of‐concept study. First, this study was conducted at a single site, which may have introduced selection bias in our results given potential demographic differences between the patient populations treated at our healthcare institution and the overall US population. Comparison with epidemiologic estimates, however, may be limited given the updated definition of IDHwt GBM in 2021 [26] and the requirement for ICI therapy in our lung cancer cohort. As patient inclusion in the analysis required multiple weight measurements, patients with more clinical follow‐up may have also been over‐selected. There also may be confounders unaccounted for among patients with GBM, such as receipt of corticosteroid therapy for control of intracranial oedema [40], which could influence weight change. Additionally, the weight values obtained in uncontrolled clinical settings may have been imprecisely measured, which may have resulted in incorrect assignment to a cachexia severity group. Finally, our DTW and clustering pipeline may be difficult to generalize to larger clinical cohorts given that the number of DTW iterations required increases at a combinatorial rate with increasing sample size.

In conclusion, we identified temporal patterns of cachexia progression and compared their ability to predict outcomes compared with an existing clinical assessment tool for cachexia. Our results suggest that prognostic value can be improved when accounting for these patterns. Recognition of concerning cachexia patterns could prompt clinicians to initiate interventions that slow the course of or redirect patients' course away from trajectories that indicate higher risk of disability, hospitalization or death—even when single cachexia marker measurements may not indicate a higher risk for poorer outcomes. Prospective identification of patient trajectories and better understanding of the mechanisms of trajectory development will be a major focus for future work to further improve upon existing systems for cachexia detection.

## Author Contributions

**Noah Forrest:** conceptualization, formal analysis, methodology, data validation, writing – original draft, writing – review and editing. **Steven Tran:** methodology. **Khizar R. Nandoliya:** data validation. **Ethan J. Houskamp:** data validation. **Tomasz Gruchala:** data validation. **Vijeeth Guggilla:** methodology. **Zequn Sun:** methodology, review and editing. **Rimas Lukas:** conceptualization, data validation, review and editing. **Derek Wainwright:** conceptualization, review and editing. **Al'ona Furmanchuk:** methodology, review and editing. **Jodi L. Johnson:** conceptualization, methodology, review and editing. **Ishan Roy:** conceptualization, formal analysis, data validation, funding acquisition, investigation, supervision, writing – original draft, writing – review and editing. **Theresa L. Walunas:** conceptualization, formal analysis, funding acquisition, investigation, methodology, resources, supervision, writing – original draft, writing – review and editing.

## Conflicts of Interest

Theresa Walunas receives unrelated research funding from Gilead Sciences.

## Supporting information


**Table S1** Case‐insensitive text searches for identification of select clinical variables
**Figure S1.** Outcome Stratification by Baselines 2015 Weight Loss Grading Scale Status
**Figure S2.** Cachexia‐free survival among 2011 consensus temporal clusters
**Figure S3.** Longitudinal WLGS Trajectories
**Table S2.** Post‐hoc inter‐cluster comparison of survival curves.
**Figure S4.** Outcome Stratification by 2015 Weight Loss Grading Scale Cluster
**Figure S5.** Inverse probability of censoring weighting using WLGS measures
**Table S3.** Hazards Ratios of Cox Proportional Hazards fit to Overall Survival
**Table S4.** Hazards Ratios of Cox Proportional Hazards fit to Overall Survival Excluding Race and Ethnicity as Covariates
**Table S5.** Hazards Ratios of Cox Proportional Hazards fit to Disability‐Free Survival
**Table S6.** Hazards Ratios of Cox Proportional Hazards fit to Disability‐Free Survival Excluding Race and Ethnicity as Covariates
**Table S7.** Hazards Ratios of Cox Proportional Hazards fit to Hospitalization‐Free Survival
**Table S8.** Hazards Ratios of Cox Proportional Hazards fit to Hospitalization‐free Survival Excluding Race and Ethnicity as Covariates
**Table S9.** Treatment Regimen Frequency Among Temporal Progression Subgroups and Overall Cohort
