# Supplementary material for: A Dynamic Time Warping Extension to Consensus Weight‐Based Cachexia Criteria Improves Prediction of Cancer Patient Outcomes
Source: JCSM Commun. 2025 Jan 29;8(1):e107. doi: 10.1002/rco2.107 (PMC11949122; doi:10.1002/rco2.107)
Supplement: Supplementary file 1 — Table S1 Case‐insensitive text searches for identification of select clinical variables Figure S1. Outcome Stratification by Baselines 2015 Weight Loss Grading Scale Status Figure S2. Cachexia‐free survival among 2011 consensus temporal clusters Figure S3. Longitudinal WLGS Trajectories Table S2. Post‐hoc inter‐cluster comparison of survival curves. Figure S4. Outcome Stratification by 2015 Weight Loss Grading Scale Cluster Figure S5. Inverse probability of censoring weighting using WLGS measures Table S3. Hazards Ratios of Cox Proportional Hazards fit to Overall Survival Table S4. Hazards Ratios of Cox Proportional Hazards fit to Overall Survival Excluding Race and Ethnicity as Covariates Table S5. Hazards Ratios of Cox Proportional Hazards fit to Disability‐Free Survival Table S6. Hazards Ratios of Cox Proportional Hazards fit to Disability‐Free Survival Excluding Race and Ethnicity as Covariates Table S7. Hazards Ratios of Cox Proportional Hazards fit to Hospitalization‐Free Survival Table S8. Hazards Ratios of Cox Proportional Hazards fit to Hospitalization‐free Survival Excluding Race and Ethnicity as Covariates Table S9. Treatment Regimen Frequency Among Temporal Progression Subgroups and Overall Cohort [file RCO2-8-e107-s001.pdf]

Supplemental Methods

Baseline Weight Loss Grading Scale (WLGS) Measures

In a similar fashion to our primary analysis using the Fearon Consensus Criteria, we first extracted each patient's maximum weight during the six months prior to initial cancer diagnosis. Next, this maximum weight was subtracted from the weight measurement that occurred closest in time relative to the initial cancer diagnosis. This difference was divided by the maximum weight to obtain the percent weight change at baseline. WLGS measures ranging from zero to four were assigned according to the BMI and weight loss matrix described in [6]. Survival analyses stratifying patients by their baseline WLGS measure is displayed in **Figure S1**.

WLGS Trajectory Identification and Characterization

To identify temporal trends of consensus criteria measures, we first extracted the same instances of weight and BMI measurements that occurred on or after a patient's cancer diagnosis described in our primary analysis. Each measurement was converted to one of five WLGS categories with values ranging from zero to four, resulting in a numeric vector of WLGS measures. The similarity of each combination of patients in the same cohort was then computed with DTW effect. All DTW distances were normalized by dividing each matrix cell by the sum of the length of the two sequences being compared to avoid dependency on the sequence length. The DTW similarity between all pairs of patients' WLGS trajectories was passed to a K-Medoids algorithm. The optimum number of subgroups, referred to as clusters, was determined visually by plotting the cluster quantity versus the average silhouette width. Trajectories were characterized identically to our method described for the primary analysis. The percentage of patients still alive and not lost to follow up with 2011 consensus criteria-defined cachexia in each WLGS trajectory subgroup was computed at one-week intervals and plotted to visualize general changes in cachexia severity over time. These trends are reported in **Figure S3**.

Predictive Capacity of WLGS Trajectories

We conducted inverse probability of censoring weighting (IPCW) to compare the predictive capacity of the WLGS-based temporal clusters versus baseline WLGS measures [38]. Patients were dichotomized as required by IPCW into cases and controls. Cases for the baseline measure were defined as a WLGS ≥ 3 or as cluster 1 (red in **Figure S3**)—the most similar trajectory to the "persistent" cluster described in the primary analysis. Comparisons of the receiver operating curves and corresponding areas under the curve were conducted at one-month intervals from the time of cancer diagnosis until the median overall survival time was reached for each cohort. Results from the IPCW analysis are reported in **Figure S5**.

Supplementary Data

Table S1. Case-insensitive text searches for identification of select clinical variables

<table>
<tr><th>Clinical Variable</th><th>Text Search (Regular Expression)</th><th>Description</th></tr>
<tr><td>Cancer Stage (Inclusion)</td><td>“(^|\s)stage(\\s+)?(IV|III|II|I)(\s+|\.|$)”</td><td>The word, “stage” preceded by a white space or found at the beginning of a document, followed by the roman numerals I, II, III, or IV. End of text must be ended with a period (end of sentence), white space (new word has started), or occur at the end of the document.</td></tr>
<tr><td>Cancer Stage (Exclusion)</td><td>"renal|neph|kidney|(^|\s)ckd|(^|\s)esrd|ulcer|breast|melanoma|endometr|prostate|colon|hepatic|liver</td><td>An inclusion instance of cancer stage was excluded if found within 100 characters of any of the included text strings. These were meant to exclude instances of other malignancies that were not lung as well as renal disease, which also uses a staging system with roman numerals.</td></tr>
<tr><td>Karnofsky Performance Status</td><td>“(\s|^)(kps|karnof)([a-zA-Z\s\%]+)?(\:|\-|\=)?(\s+)?\d+”</td><td>The text string “kps” or “karnof…” preceded by a white space or found at the beginning of a document; followed by an optional colon, dash, or equal sign; an optional white space; and ended by a digit (between 0 and 100 after further filtering).</td></tr>
<tr><td>Eastern Cooperative Oncology Group Performance Status</td><td>“(\s|^)(ecog|eastern\s+cooperative)([a-zA-Z\s]+|\/WHO)?(\:|\-|\=)?(\s+)?(\d+|zero|one|two|three|four|five)”</td><td>The text string “ecog” or “eastern…cooperative” or “who”; preceded by a white space or found at the beginning of a document; followed by an optional dash, colon, or equal sign; an optional white space; and ended by a digit or the words representing numbers one through five.</td></tr>
</table>

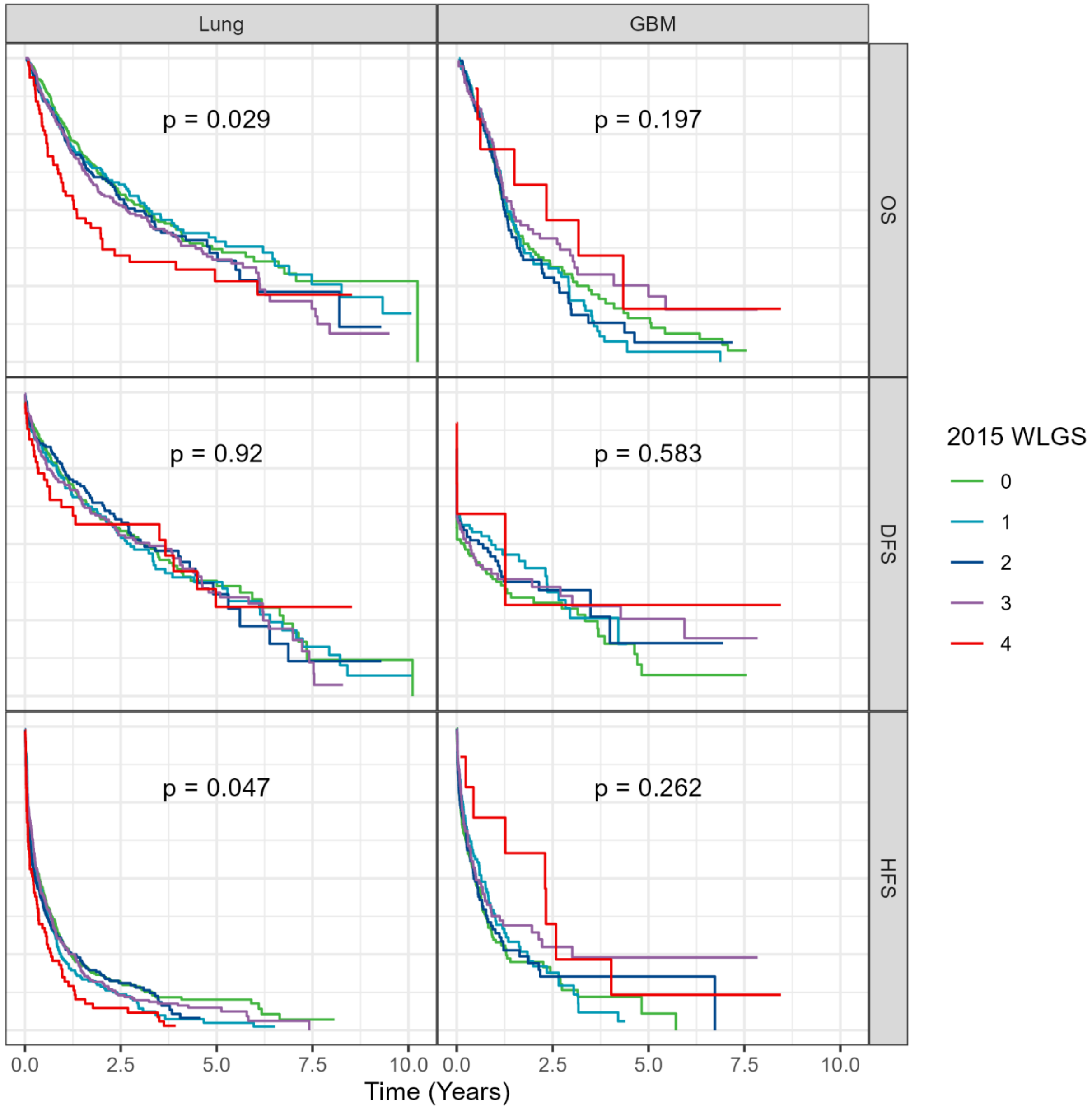

Fig S1. Outcome Stratification by Baselines 2015 Weight Loss Grading Scale Status

Footnote: Patients were stratified by their WLGS measure at the weight measure closest in time to cancer diagnosis in an analogous fashion to the 2011 Consensus Criteria. Lung = Patients with a billed diagnosis of lung cancer and treatment with immune checkpoint inhibitor therapy; WLGS = Weight loss grading scale; OS = overall survival; DFS = disability-free survival; HFS = hospitalization-free survival

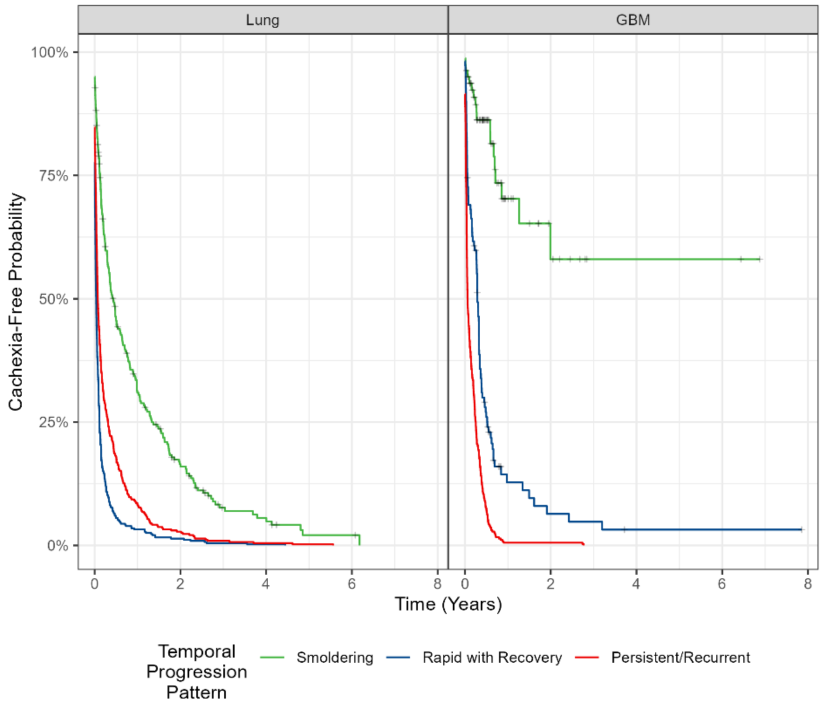

Fig S2. Cachexia-free survival among 2011 consensus temporal clusters

The time to patients' first Consensus Criteria measurement of cachexia was computed. Time-to-event curves were constructed stratified by the temporal progression patterns identified via DTW and k-medoids clustering. Patients were censored if death occurred prior to cachexia development.

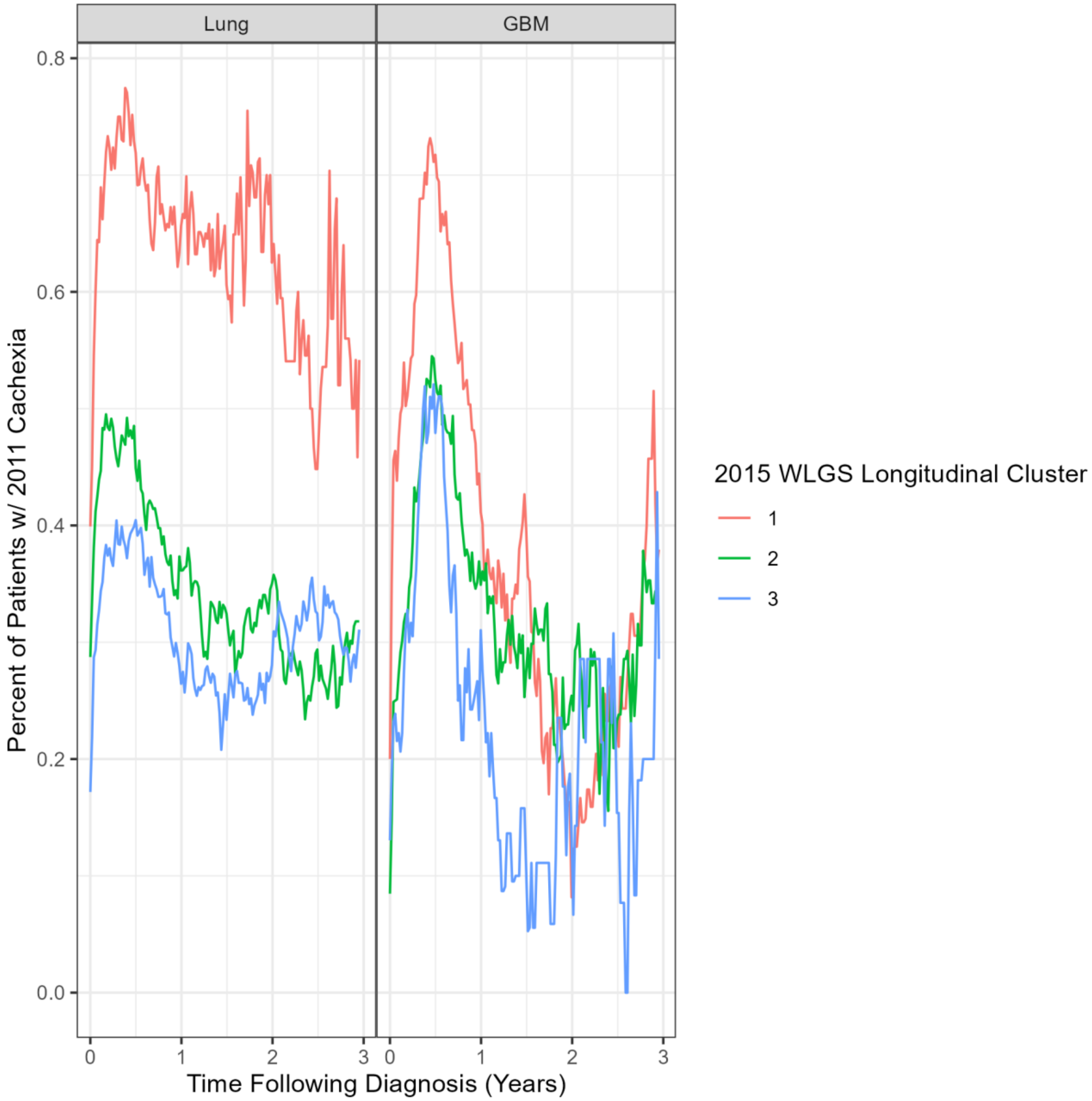

Fig S3. Longitudinal WLGS Trajectories

Patients' longitudinal 2015 WLGS measures were clustered using dynamic time warping and k-medoids clustering in an analogous fashion to the longitudinal 2011 Consensus Criteria measures. The percent of patients still alive with 2011 Consensus Criteria-defined cachexia at one-week intervals was computed out to 3 years following initial cancer diagnosis. WLGS = Weight Loss Grading Scale.

Table S2. Post-hoc inter-cluster comparison of survival curves .

			Outcome		
**Cohort**	**Cluster A[a]**	**Cluster B[b]**	**OS**	**DFS**	**HFS**
GBM	Persistent/Recurrent	Smoldering	0.44	0.27	0.0019
GBM	Persistent/Recurrent	Rapid/Recovery	<0.0001	0.0014	0.054
GBM	Smoldering	Rapid/Recovery	<0.0001	0.17	0.15
Lung	Persistent/Recurrent	Smoldering	<0.0001	<0.0001	<0.0001
Lung	Persistent/Recurrent	Rapid/Recovery	<0.0001	<0.0001	0.075
Lung	Smoldering	Rapid/Recovery	<0.0001	<0.0001	0.00019

P values determined using log rank test with built in R package functions. OS, Overall survival; DFS, disability-free survival; HFS, hospitalization-free survival; GBM, Glioblastoma.

a First cluster being compared

b second cluster being compared

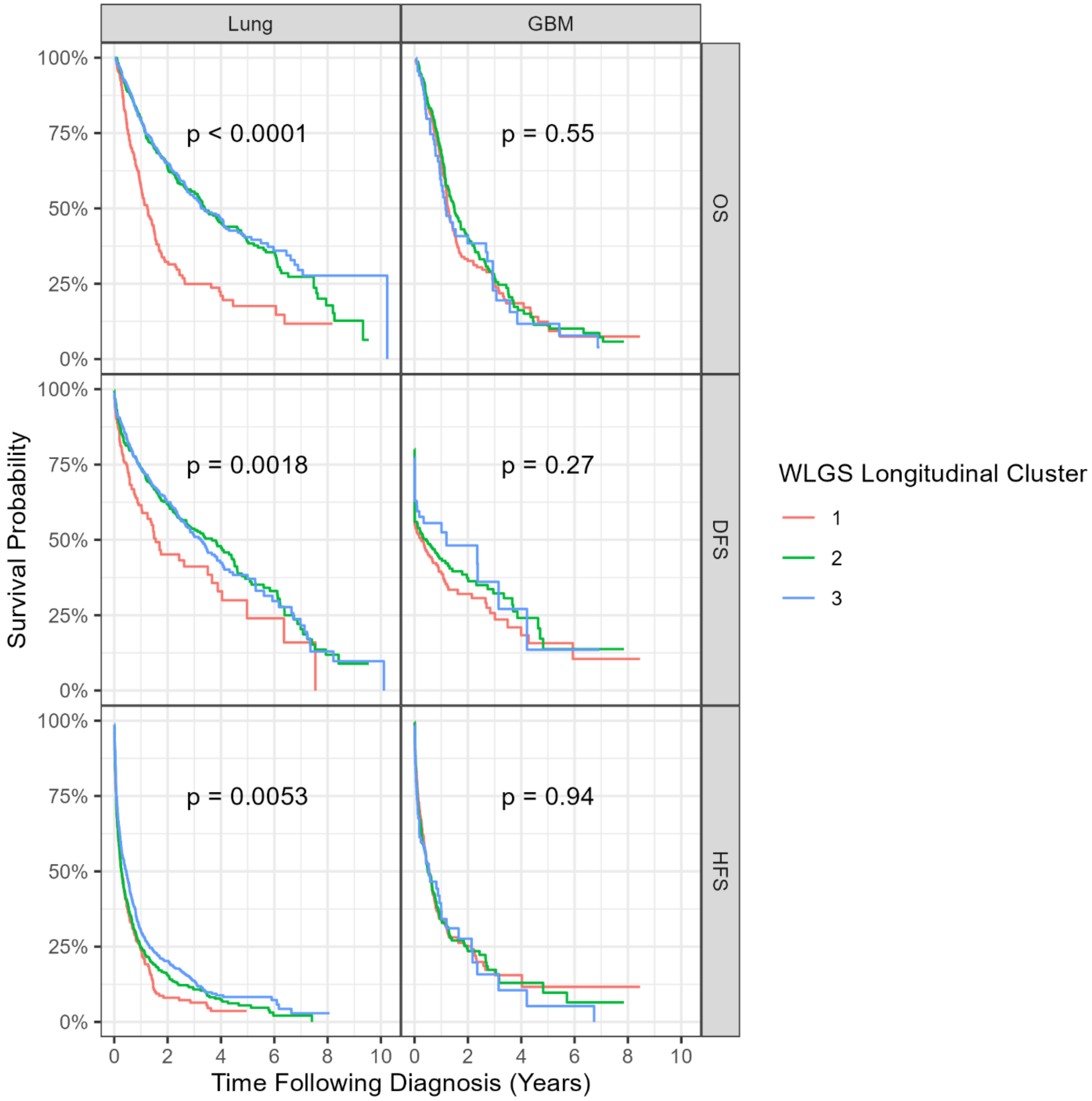

Fig S4. Outcome Stratification by 2015 Weight Loss Grading Scale Cluster

Patients were stratified by the longitudinal trajectory clusters that utilized 2015 WLGS measures (See Fig S2) for Kaplan-Meier analysis in an analogous fashion to that reported in Figure 3 that stratified patients by the longitudinal patients by clusters defined by longitudinal 2011 consensus criteria measures.

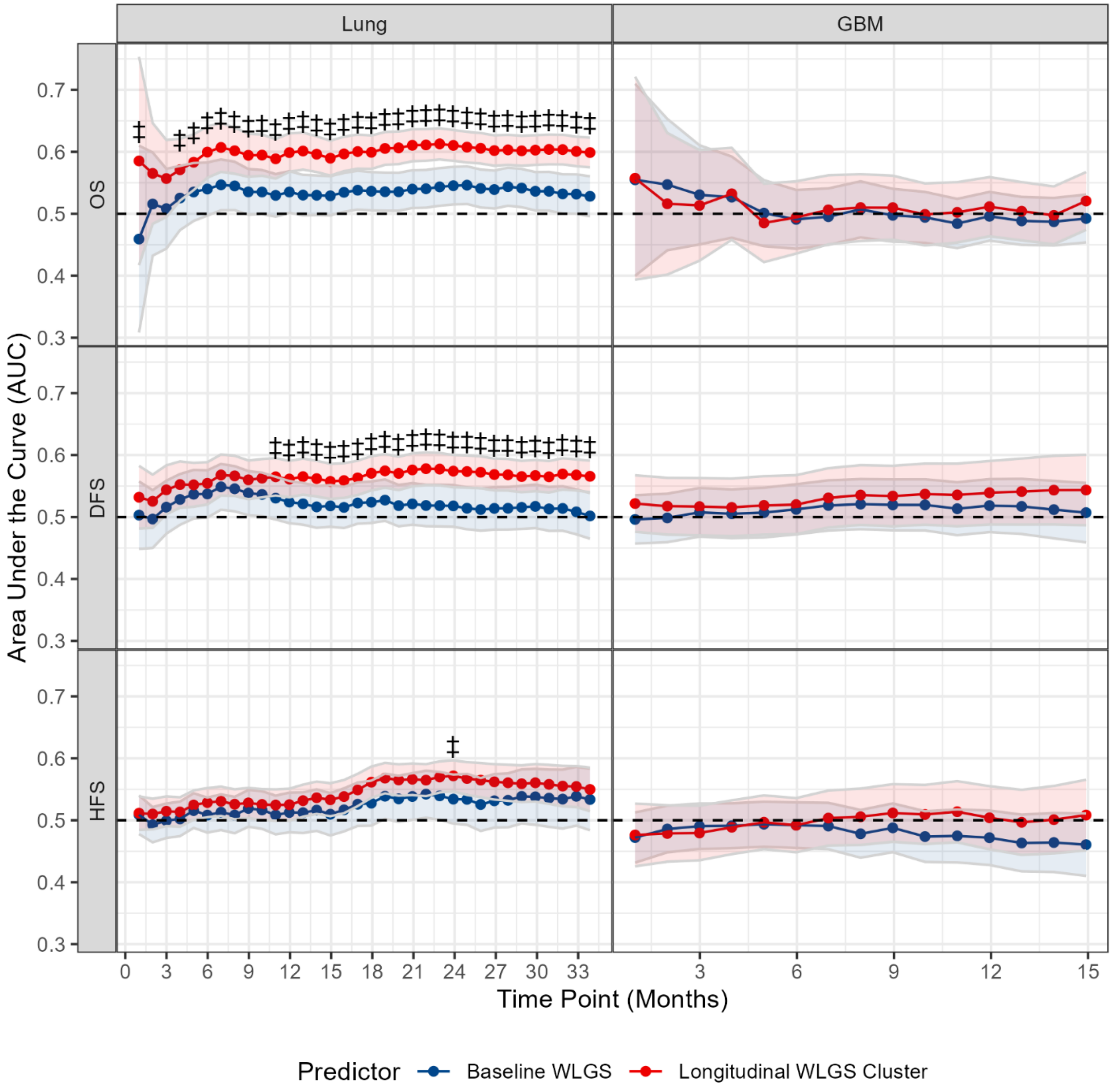

Fig S5. Inverse probability of censoring weighting using WLGS measures

In an analogous fashion to that reported in Fig 5, areas under the curve (AUC) for the baseline WLGS measure and temporal cachexia patterns determined through DTW and clustering of longitudinal WLGS measures were computed at one-month intervals starting at the time of cancer diagnosis until the time of median overall survival was reached for each cohort. Values were compared between the baseline WLGS measure versus temporal clusters to identify differences in cachexia measure performance in stratifying patient outcomes. The AUC describes the frequency with which the higher risk group—defined as a WLGS measure ≥3 for the baseline measurement and cluster 1 for the temporal cluster (see Fig S3). Higher AUC values indicate variables that more effectively stratify patient outcomes.

OS, Overall Survival; DFS, Disability-free survival; HFS, Hospitalization-free survival, Shaded region, confidence interval at the 95% confidence level; ‡ Significant difference between receiver operating curves of baseline and temporal cluster Kaplan-Meier predictions at indicated time point and 95% confidence level.

Table S3. Hazards Ratios of Cox Proportional Hazards fit to Overall Survival

				Univariate	Multivariate Baseline Consensus	Multivariate Temporal Cluster
	**Variable**	**Referent Level**	**Non-Referent Level**			
**Lung**	Baseline Consensus[a]	Not cachexia	Cachexia	1.5 (1.2-1.7)*	1.4 (1.2-1.7)*	-
	Temporal Cluster[b]	Not Persistent or Recurrent	Persistent or Recurrent	4.9 (4.1-5.9)*	-	4.8 (4.1-5.8)*
	Sex	F	M	1.3 (1.1-1.5)*	1.3 (1.1-1.6)*	1.4 (1.2-1.7)*
	Race	White	Non-White	0.84 (0.7-1)	0.85 (0.69-1.0)	0.81 (0.66-1)
	Ethnic Group	Not Hispanic	Hispanic	1.3 (0.85-2.1)	1.3 (0.8-2.0)	1.3 (0.8-2)
	Age Group	18-64	65 and Up	1.1 (0.89-1.2)	1.1 (0.94-1.3)	1 (0.88-1.2)
	ECOG	0	>0	1.4 (1.2-1.7)*	1.4 (1.2-1.7)*	1.3 (1.1-1.6)*
	Cancer Stage	I-III	IV	1.6 (1.4-1.9)*	1.7 (1.4-2.0)*	1.6 (1.3-1.8)*
**GBM**	Baseline Consensus	Not cachexia	Cachexia	0.9 (0.7-1.1)	0.9 (0.8-1.2)	-
	Temporal Cluster	Not Persistent or Recurrent	Persistent or Recurrent	2.3 (1.8-2.9)*	-	1.9 (1.4-2.4)*
	Sex	F	M	1.2 (1-1.5)*	1.2 (0.9-1.5)	1.2 (0.9-1.4)
	Race	White	Non-White	0.8 (0.6-1.1)	0.73 (0.6-0.98)*	0.7 (0.5-0.9)*
	Ethnic Group	Not Hispanic	Hispanic	0.8 (0.5-1.2)	0.8 (0.5-1.3)	0.8 (0.5-1.3)
	Age Group	18-64	65 and Up	1.9 (1.5-2.3)*	2.1 (1.7-2.6)*	1.9 (1.5-2.3)*
	KPS	KPS >=90	KPS < 90	1.8 (1.5-2.3)*	1.9 (1.5-2.3)*	1.7 (1.4-2.1)*
	Anatomic Location[c]	Unilateral Cortical	Progressive Tumor	1.5 (1.2-2)*	1.7 (1.3-2.2)*	1.6 (1.2-2.1)*
	MGMT Promoter	Not Unmethylated	Unmethylated	2 (1.6-2.4)*	2.1 (1.7-2.7)*	2.1 (1.7-2.6)*

ECOG, Eastern Cooperative Oncology Group scale; KPS, Karnofsky Performance Status Scale; MGMT Promoter, O6-methylguanine-DNA-methyltransferase promoter status; GBM, Glioblastoma.

* = HR p value for model coefficient < 0.05

a. consensus criteria category at the time of cancer diagnosis, dichotomized

b. cachexia progression pattern as determined by dynamic time warping and subsequent clustering

c. dichotomized variable, where progressive tumor is defined as multifocal or non-cortical tumors

Table S4. Hazards Ratios of Cox Proportional Hazards fit to Overall Survival Excluding Race and Ethnicity as Covariates

				**Multivariate Baseline Consensus**	**Multivariate Temporal Cluster**
	**Variable**	**Referent Level**	**Non-Referent Level**		
**Lung**	Baseline Consensus[a]	Not cachexia	Cachexia	1.4 (1.2-1.7)*	-
	Temporal Cluster[b]	Not Persistent or Recurrent	Persistent or Recurrent	-	4.8 (4.0-5.8)*
	Sex	F	M	1.4 (1.2-1.6)*	1.4 (1.2-1.7)*
	Age Group	18-64	65 and Up	1.1 (0.95-1.3)	1.1 (0.89-1.3)
	ECOG	0	>0	1.4 (1.2-1.6)*	1.3 (1.1-1.6)*
	Cancer Stage	I-III	IV	1.7 (1.4-2.0)*	1.6 (1.3-1.8)*
**GBM**	Baseline Consensus	Not cachexia	Cachexia	0.95 (0.77-1.2)	-
	Temporal Cluster	Not Persistent or Recurrent	Persistent or Recurrent	-	1.8 (1.4-2.2)*
	Sex	F	M	1.2 (0.96-1.5)	1.2 (0.96-1.5)
	Age Group	18-64	65 and Up	2.1 (1.7-2.6)*	1.9 (1.5-2.4)*
	KPS	KPS >=90	KPS < 90	1.8 (1.4-2.2)*	1.6 (1.3-2.)*
	Anatomic Location[c]	Unilateral Cortical	Progressive Tumor	1.7 (1.3-2.2)*	1.6 (1.2-2.1)*
	MGMT Promoter	Not Unmethylated	Unmethylated	2.1 (1.7-2.6)*	2.1 (1.7-2.6)*

ECOG, Eastern Cooperative Oncology Group scale; KPS, Karnofsky Performance Status Scale; MGMT Promoter, O6-methylguanine-DNA-methyltransferase promoter status; GBM, Glioblastoma.

* = HR p value for model coefficient < 0.05

a. consensus criteria category at the time of cancer diagnosis, dichotomized

b. cachexia progression pattern as determined by dynamic time warping and subsequent clustering

c. dichotomized variable, where progressive tumor is defined as multifocal or non-cortical tumors

Table S5. Hazards Ratios of Cox Proportional Hazards fit to Disability-Free Survival

				Univariate	Multivariate Baseline Consensus	Multivariate Temporal Cluster
	**Variable**	**Referent Level**	**Non-Referent Level**			
**Lung**	Baseline Consensus[a]	Not cachexia	Cachexia	1.3 (1.1-1.7)*	1.3 (1.1-1.6)*	-
	Temporal Cluster[b]	Not Persistent or Recurrent	Persistent or Recurrent	2.1 (1.7-2.6)*	-	2 (1.7-2.5)*
	Sex	F	M	1.1 (0.9-1.4)	1.1 (1-1.4)	1.1 (0.9-1.4)
	Race	White	Non-White	1.1 (0.9-1.4)	1.1 (0.9-1.4)	1.1 (0.9-1.3)
	Ethnic Group	Not Hispanic	Hispanic	1.5 (0.9-2.4)	1.5 (0.9-2.4)	1.5 (1.0-2.5)
	Age Group	18-64	65 and Up	1.2 (1.0-1.4)	1.3 (1.0-1.5)*	1.2 (1.0-1.5)*
	ECOG	0	>0	1.4 (1.2-1.7)*	1.4 (1.1-1.6)*	1.3 (1.1-1.6)*
	Cancer Stage	I-III	IV	1.2 (1.0-1.4)	1.2 (1.0-1.4)	1.1 (1.0-1.4)
**GBM**	Baseline Consensus	Not cachexia	Cachexia	0.9 (0.8-1.2)	0.9 (0.7-1.2)	-
	Temporal Cluster	Not Persistent or Recurrent	Persistent or Recurrent	1.4 (1.1-1.8)*	-	1.3 (1.01-1.7)*
	Sex	F	M	0.9 (0.8-1.2)	0.9 (0.7-1.2)	0.9 (0.7-1.2)
	Race	White	Non-White	0.9 (0.6-1.2)	0.84 (0.6-1.1)	0.83 (0.6-1.1)
	Ethnic Group	Not Hispanic	Hispanic	0.8(0.5-1.3)	0.87 (0.5-1.5)	0.84 (0.5-1.4)
	Age Group	18-64	65 and Up	1.4 (1.1-1.8)*	1.4 (1.1-1.8)*	1.4 (1.1-1.8)*
	KPS	KPS >=90	KPS < 90	1.2 (1.0-1.5)	1.2 (1.0-1.5)	1.1 (0.9-1.4)
	Anatomic Location[c]	Unilateral Cortical	Progressive Tumor	1.2 (0.9-1.6)	1.2 (0.9-1.7)	1.2 (0.8-1.6)
	MGMT Promoter	Not Unmethylated	Unmethylated	1.0 (0.8-1.3)	1.0 (0.8-1.3)	1.0 (0.8-1.3)

ECOG, Eastern Cooperative Oncology Group scale; KPS, Karnofsky Performance Status Scale; MGMT Promoter, O6-methylguanine-DNA-methyltransferase promoter status; GBM, Glioblastoma.

* = HR p value for model coefficient < 0.05

a. consensus criteria category at the time of cancer diagnosis, dichotomized

b. cachexia progression pattern as determined by dynamic time warping and subsequent clustering

c. dichotomized variable, where progressive tumor is defined as multifocal or non-cortical tumors

Table S6. Hazards Ratios of Cox Proportional Hazards fit to Disability-Free Survival Excluding Race and Ethnicity as Covariates

				Multivariate Baseline Consensus	Multivariate Temporal Cluster
	**Variable**	**Referent Level**	**Non-Referent Level**		
**Lung**	Baseline Consensus[a]	Not cachexia	Cachexia	1.3 (1.1-1.6)*	-
	Temporal Cluster[b]	Not Persistent or Recurrent	Persistent or Recurrent	-	4.8 (4.0-5.8)*
	Sex	F	M	1.1 (0.95-1.4)	1.1 (0.94-1.3)
	Age Group	18-64	65 and Up	1.2 (1.0-1.5)*	1.2 (1.0-1.5)*
	ECOG	0	>0	1.4 (1.1-1.7)*	1.4 (1.1-1.6)*
	Cancer Stage	I-III	IV	1.2 (0.98-1.4)	1.1 (0.96-1.4)*
**GBM**	Baseline Consensus	Not cachexia	Cachexia	0.92 (0.73-1.2)	-
	Temporal Cluster	Not Persistent or Recurrent	Persistent or Recurrent	-	1.3 (1.0-1.7)*
	Sex	F	M	0.93 (0.74-1.2)	0.94 (0.75-1.2)
	Age Group	18-64	65 and Up	1.5 (1.1-1.8)*	1.4 (1.1-1.8)*
	KPS	KPS >=90	KPS < 90	1.2 (0.93-1.5)	1..1 (0.88-1.4)
	Anatomic Location[c]	Unilateral Cortical	Progressive Tumor	1.2 (0.89-1.7)	1.2 (0.84-1.6)
	MGMT Promoter	Not Unmethylated	Unmethylated	1.0 (0.8-1.3)	1.3 (1.0-1.7)*

ECOG, Eastern Cooperative Oncology Group scale; KPS, Karnofsky Performance Status Scale; MGMT Promoter, O6-methylguanine-DNA-methyltransferase promoter status; GBM, Glioblastoma.

* = HR p value for model coefficient < 0.05

a. consensus criteria category at the time of cancer diagnosis, dichotomized

b. cachexia progression pattern as determined by dynamic time warping and subsequent clustering

c. dichotomized variable, where progressive tumor is defined as multifocal or non-cortical tumors

Table S7. Hazards Ratios of Cox Proportional Hazards fit to Hospitalization-Free Survival

				**Univariate**	**Multivariate Baseline Consensus**	**Multivariate Temporal Cluster**
**Lung**	**Variable**	**Referent Level**	**Non-Referent Level**			
	Baseline Consensus[a]	Not cachexia	Cachexia	1.3 (1.2-1.5)*	1.3 (1.1-1.5)*	-
	Temporal Cluster[b]	Not Persistent or Recurrent	Persistent or Recurrent	1.3 (1.1-1.5)*	-	1.3 (1.1-1.4)*
	Sex	F	M	1.2 (1.0-1.3)*	1.2 (1.0-1.3)*	1.2 (1-1.3)*
	Race	White	Non-White	1.1 (0.9-1.3)	1.1 (1.0-1.3)	1.1 (0.95-1.3)
	Ethnic Group	Not Hispanic	Hispanic	1.1 (0.8-1.6)	1.1 (0.8-1.6)	1.1 (0.76-1.6)
	Age Group	18-64	65 and Up	1.1 (0.9-1.2)	1.1 (1.0-1.2)	1.1 (0.93-1.2)
	ECOG	0	>0	1.2 (1.1-1.4)*	1.2 (1.01-1.3)*	1.2 (1-1.3)*
	Cancer Stage	I-III	IV	1.1 (1.0-1.3)	1.1 (0.99-1.3)	1.1 (0.98-1.3)
**GBM**	Baseline Consensus	Not cachexia	Cachexia	1.2 (1.0-1.4)	1.2 (0.99-1.5)	-
	Temporal Cluster	Not Persistent or Recurrent	Persistent or Recurrent	1.4 (1.1-1.8)*	-	1.3 (1-1.6)*
	Sex	F	M	1.1 (0.9-1.4)	1.1 (0.9-1.4)	1.1 (0.89-1.3)
	Race	White	Non-White	0.9 (0.7-1.1)	0.9 (0.7-1.1)	0.82 (0.62-1.1)
	Ethnic Group	Not Hispanic	Hispanic	0.9 (0.6-1.4)	0.83 (0.5-1.3)	0.89 (0.57-1.4)
	Age Group	18-64	65 and Up	1.3 (1.1-1.6)*	1.3 (1.1-1.6)*	1.3 (1.1-1.6)*
	KPS	KPS >=90	KPS < 90	1.4 (1.2-1.7)*	1.4 (1.1-1.7)*	1.3 (1.1-1.7)*
	Anatomic Location[c]	Unilateral Cortical	Progressive Tumor	1.4 (1-1.8)*	1.4 (1.1-1.8)*	1.4 (1-1.8)*
	MGMT Promoter	Not Unmethylated	Unmethylated	1.5 (1.2-1.9)*	1.6 (1.3-2.0)*	1.5 (1.2-1.9)*

ECOG, Eastern Cooperative Oncology Group scale; KPS, Karnofsky Performance Status Scale; MGMT Promoter, O6-methylguanine-DNA-methyltransferase promoter status; GBM, Glioblastoma.

* = HR p value for model coefficient < 0.05

a. consensus criteria category at the time of cancer diagnosis, dichotomized

b. cachexia progression pattern as determined by dynamic time warping and subsequent clustering of 2011 consensus criteria measures

c. dichotomized variable, where progressive tumor is defined as multifocal or non-cortical tumors

Table S8. Hazards Ratios of Cox Proportional Hazards fit to Hospitalization-free Survival Excluding Race and Ethnicity as Covariates

				**Multivariate Baseline Consensus**	**Multivariate Temporal Cluster**
**Lung**	**Variable**	**Referent Level**	**Non-Referent Level**		
	Baseline Consensus[a]	Not cachexia	Cachexia	1.3 (1.1-1.5)*	-
	Temporal Cluster[b]	Not Persistent or Recurrent	Persistent or Recurrent	-	1.3 (1.1-1.4)*
	Sex	F	M	1.2 (1-1.3)*	1.2 (1-1.3)*
	Age Group	18-64	65 and Up	1.1 (0.94-1.2)	1.1 (0.93-1.2)
	ECOG	0	>0	1.2 (1.0-1.3)*	1.2 (1-1.3)*
	Cancer Stage	I-III	IV	1.1 (0.98-1.3)	1.1 (0.97-1.3)
**GBM**	Baseline Consensus	Not cachexia	Cachexia	1.2 (0.99-1.5)	-
	Temporal Cluster	Not Persistent or Recurrent	Persistent or Recurrent	-	1.3 (1.0-1.6)*
	Sex	F	M	1.1 (0.91-1.4)	1.1 (0.97-1.3)
	Age Group	18-64	65 and Up	1.3 (1.1-1.6)*	1.3 (1.1-1.6)*
	KPS	KPS >=90	KPS < 90	1.4 (1.1-1.7)*	1.3 (1.1-1.6)*
	Anatomic Location[c]	Unilateral Cortical	Progressive Tumor	1.4 (1.1-1.8)	1.4 (1.0-1.8)*
	MGMT Promoter	Not Unmethylated	Unmethylated	1.6 (1.3-1.9)*	1.5 (1.2-1.9)*

ECOG, Eastern Cooperative Oncology Group scale; KPS, Karnofsky Performance Status Scale; MGMT Promoter, O6-methylguanine-DNA-methyltransferase promoter status; GBM, Glioblastoma.

* = HR p value for model coefficient < 0.05

a. consensus criteria category at the time of cancer diagnosis, dichotomized

b. cachexia progression pattern as determined by dynamic time warping and subsequent clustering

c. dichotomized variable, where progressive tumor is defined as multifocal or non-cortical tumors

Table S9. Treatment Regimen Frequency Among Temporal Progression Subgroups and Overall Cohort

		Temporal Progression Group			Overall Cohort
	**Treatment Regimen**	**Persistent**	**Smoldering**	**Rapid with Recovery**	
		**N = 429**	**N = 263**	**N = 431**	**N = 1,123**
		**N (%)**	**N (%)**	**N (%)**	**N (%)**
Lung	Pembrolizumab	181 (43)	89 (34)	179 (42)	449 (41)
	Nivolumab	78 (19)	48 (19)	77 (18)	203 (18)
	Atezolizumab	58 (14)	34 (13)	58 (14)	150 (13)
	Ipilimumab	0 (0)	0 (0)	1 (<1)	1 (<1)
	Durvalumab	20 (5)	30 (12)	35 (9)	85 (8)
	Other ICI[a]	37 (9)	5 (2)	9 (3)	51 (5)
	Combination of ICIs[b]	55 (13)	57 (22)	72 (17)	184 (16)
GBM		**N = 355**	**N = 80**	**N = 110**	**N = 545**
		**N (%)**	**N (%)**	**N (%)**	**N (%)**
	No RT nor TMZ	25 (7)	13 (16)	7 (6)	45 (8)
	RT Only	13 (4)	3 (4)	4 (4)	20 (4)
	TMZ Only	64 (18)	21 (26)	18 (16)	103 (19)
	RT and TMZ	253 (71)	43 (54)	81 (74)	377 (69)

ICI therapy type and the presence of TMZ were obtained through text searches of medication orders in the electronic health record data of patients in each cohort. RT was identified via text searches of procedure orders in the electronic health record of patients with GBM.

ICI, Immune checkpoint Inhibitor; RT, Radiotherapy; TMZ, Temozolomide; GBM, Glioblastoma

a. Any ICI not included in Pembrolizumab, Nivolumab, Atezolizumab, Ipilimumab, or Durvalumab.
b. Any combination of two or more of five named ICIs
